# ASD Is Not DLI: Individuals With Autism and Individuals With Syntactic DLI Show Similar Performance Level in Syntactic Tasks, but Different Error Patterns

**DOI:** 10.3389/fpsyg.2018.00279

**Published:** 2018-04-04

**Authors:** Nufar Sukenik, Naama Friedmann

**Affiliations:** Language and Brain Lab, School of Education, Tel Aviv University, Tel Aviv, Israel

**Keywords:** ASD, SLI, syntax, relative clauses, syntactic impairment

## Abstract

Do individuals with autism have a developmental syntactic impairment, DLI (formerly known as SLI)? In this study we directly compared the performance of 18 individuals with Autism Spectrum Disorder (ASD) aged 9;0–18;0 years with that of 93 individuals with Syntactic-Developmental Language Impairment (SyDLI) aged 8;8–14;6 (and with 166 typically-developing children aged 5;2–18;1). We tested them using three syntactic tests assessing the comprehension and production of syntactic structures that are known to be sensitive to syntactic impairment: elicitation of subject and object relative clauses, reading and paraphrasing of object relatives, and repetition of complex syntactic structures including Wh questions, relative clauses, topicalized sentences, sentences with verb movement, sentences with A-movement, and embedded sentences. The results were consistent across the three tasks: the overall rate of correct performance on the syntactic tasks is similar for the children with ASD and those with SyDLI. However, once we look closer, they are very different. The types of errors of the ASD group differ from those of the SyDLI group—the children with ASD provide various types of pragmatically infelicitous responses that are not evinced in the SyDLI or in the age equivalent typically-developing groups. The two groups (ASD and SyDLI) also differ in the pattern of performance—the children with SyDLI show a syntactically-principled pattern of impairment, with selective difficulty in specific sentence types (such as sentences derived by movement of the object across the subject), and normal performance on other structures (such as simple sentences). In contrast, the ASD participants showed generalized low performance on the various sentence structures. Syntactic performance was far from consistent within the ASD group. Whereas all ASD participants had errors that can originate in pragmatic/discourse difficulties, seven of them had completely normal syntax in the structures we tested, and were able to produce, understand, and repeat relative clauses, Wh questions, and topicalized sentences. Only one ASD participant showed a syntactically-principled deficit similar to that of individuals with SyDLI. We conclude that not all individuals with ASD have syntactic difficulties, and that even when they fail in a syntactic task, this does not necessarily originate in a syntactic impairment. This shows that looking only at the total score in a syntactic test may be insufficient, and a fuller picture emerges once the performance on different structures and the types of erroneous responses are analyzed.

## Introduction

Autism Spectrum Disorder (ASD) is characterized by a triad of impairments that affect communication, social interaction, and behavioral repertoire (Pickles et al., 2009; Loucas et al., 2013). One of the most debated issues in the research on language abilities in children with ASD is whether their communication difficulties are a result of language impairment, with characteristics similar to those found in non-ASD children with Developmental Language Impairment (DLI, previously known as “SLI”^1^). This is the question we examine in this study, by directly comparing the performance of individuals with ASD to that of individuals with DLI. We focus on a specific type of DLI that selectively affects syntax, Syntactic DLI (SyDLI for short), and compare the performance of the two groups in syntactic tests of comprehension, production, and repetition of complex syntactic structures.

The differences and possible overlap between DLI and ASD captured the interest of many researchers over the last decade (see Bishop, 2003; Botting and Conti-Ramsden, 2003; Tager-Flusberg, 2006; Eigsti and Bennetto, 2009; Tomblin, 2011; Terzi et al., 2014; Tuller et al., 2017, for extensive reviews). Studies of this question tested various language domains and reached different conclusions. Several studies examined the similarity between ASD and DLI in the lexical domain. McGregor et al. (2012) and Demouy et al. (2011) both found that the ASD groups they tested showed similar lexical performance to that of the DLI group, but the error patterns differed between the groups. Both studies reported that the ASD group committed more pragmatic errors than the DLI group (McGregor et al. also reported the same for the ASD group in comparison to the age-matched TD group). Different results were found in other studies, which reported that individuals with ASD performed more poorly than those with DLI on input lexical tasks (Loucas et al., 2013), and in linguistic concept tests (Manolitsi and Botting, 2011). Yet others found that individuals with ASD were better than those with DLI in tasks such as word association and word structure (Lloyd et al., 2006).

Other studies examined phonological abilities in ASD, in comparison to DLI, mostly through the repetition of nonwords (Whitehouse et al., 2008; Demouy et al., 2011; Riches et al., 2011; Williams et al., 2013). In this domain, too, the results are mixed. Demouy et al. (2011), Whitehouse et al. (2008), and Loucas et al. (2010) reported that children with ASD who had language impairment (ASD+LI; LI defined as scores below the norms on standardized language tasks) showed impaired performance in nonword repetition that was similar to that of the DLI group and lower than TD. In contrast, Durrleman and Delage (2016) and Riches et al. (2011) reported that the nonword repetition of the ASD (who had LI) participants was better than that of the DLI participants.

This distinction, which was made in various studies, between ASD with language impairment and ASD with normal language (e.g., Kjelgaard and Tager-Flusberg, 2001; Whitehouse et al., 2008; McGregor et al., 2012; Gavarró and Heshmati, 2014; Modyanova et al., 2017; Tuller et al., 2017) reflects an important insight with respect to language in ASD. This is also a conclusion of various studies comparing ASD to DLI: some ASD participants show language impairment, whereas others have language performance similar to TD (see e.g., Kjelgaard and Tager-Flusberg, 2001). Kjelgaard and Tager-Flusberg (2001) also report another subgroup of ASD participants, who show language difficulties across all language structures and domains they tested. Such studies indicate that it might be impossible to make a general claim regarding language in ASD, given the considerable heterogeneity within this group (Lombardo et al., 2015; see also Brock et al., 2017).

Thus, the heterogeneity of impairment within the ASD group may be one source of the differences between the results of different studies that examined whether the language difficulty in ASD resembles the language impairment in DLI. Another source is the heterogeneity within the DLI group. Several studies of DLI showed that DLI has many faces, and that various language domains can be selectively affected, giving rise to various types of DLI, which selectively affect syntax, lexicon, phonology, or pragmatics (Korkman and Hakkinen-Rihu, 1994; Conti-Ramsden et al., 1997, 2001, 2006; Conti-Ramsden and Botting, 1999; van Daal et al., 2004; Bishop, 2006; Friedmann and Novogrodsky, 2008, 2011).

One such type of DLI, which has been studied extensively, is Syntactic DLI (or SyDLI), in which the syntactic abilities are specifically affected. The main syntactic constructs that have been identified as impaired in SyDLI are structures that involve syntactic movement (Adams, 1990; van der Lely and Harris, 1990; Håkansson and Hansson, 2000; Schuele and Tolbert, 2001; Stavrakaki, 2001; Friedmann and Novogrodsky, 2004, 2007, 2011; Hamann, 2005; Novogrodsky and Friedmann, 2006; Jakubowicz and Gutierrez, 2007; Levy and Friedmann, 2009; Jakubowicz, 2011; Friedmann et al., 2015; Hamann and Tuller, 2015); pronominal object clitics (Jakubowicz et al., 1998; Hamann et al., 2003; Paradis et al., 2003; Hamann, 2004; Parisse and Maillart, 2004; Jakubowicz and Tuller, 2008; Stavrakaki et al., 2011; Tuller et al., 2011), and verb inflections (Wexler, 2011; Rothweiler et al., 2012; Leonard, 2017).

These syntactic domains that have been identified as clinical markers for syntactic impairment in DLI are the best targets for examining whether ASD resembles DLI. Indeed, these domains have been tested in ASD, again, with mixed results. Terzi et al. (2014) tested structures that are considered clinical markers for DLI in Greek—passive sentences, pronouns, and pronominal clitics—in Greek-speaking children with ASD aged 5–8 years. They found that the children with ASD performed similarly to TD children in passive sentences and pronouns, but poorer than the TD children in the comprehension of pronominal clitics. Whitehouse et al. (2008) compared the performance of English-speaking ASD+LI group to a DLI group in the TROG (Test for reception of grammar, Bishop, 1989) sentence comprehension and sentence repetition task. They reported that the ASD+LI participants performed similarly to the DLI on the sentence comprehension task but better than the DLI on sentence repetition. Manolitsi and Botting (2011), who also compared comprehension and production in ASD and DLI, reached a different conclusion: the children with ASD they tested performed poorer than the DLI on receptive language tasks and similar to DLI in sentence production tasks. Roberts et al. (2004) tested the performance of English-speaking children with ASD on 3rd person- and past tense morphology. They report that a subgroup of the ASD group who had a language impairment showed high rates of omissions of tense marking, like English-speaking children with DLI.

Roberts et al. also noticed an important difference between the populations with respect to the types of incorrect responses they produced. The children with ASD made errors that the DLI did not make, such as echolalic responses and perseverations, as well as semantically inappropriate, off-topic responses (see also Modyanova et al., 2017, for error types that they termed “unscorable”, which the ASD+LI group makes but the DLI and the TD groups do not). The same point regarding different error types was also made by Demouy et al. (2011), who assessed sentence comprehension and production in French-speaking ASD and DLI participants. They found that the ASD group performed similarly to the DLI group in comprehension, and that both groups showed impaired performance in sentence production. However, the groups crucially differed with respect to the errors they made: the children with ASD produced significantly more pragmatic errors than the DLI participants. Such pragmatic errors are responses that are inappropriate, unrelated to the stimuli, reflecting misunderstanding of the situation in the stimuli or failing to understand the intention of the experimenter and the purpose of the conversation.

Beyond the important finding that children with ASD make errors that other language impaired children do not, researchers noticed that the pattern of performance across different sentence types also differs. Gavarró and Heshmati (2014) tested the comprehension of passive sentences in Persian-speaking children with ASD. They found that a subgroup of the ASD (classified as low-functioning ASD) performed poorly on these structures. An important observation these researchers made was that the children with ASD who made errors in this task actually performed poorly on all sentence structures, including active sentences, unlike children with DLI, who are selectively impaired in passive sentences, but not active sentences (e.g., van der Lely, 1996, for English passives in DLI). Durrleman et al. (2017) also tested various types of passives vs. active sentences in ASD and also found that children with ASD performed poorly on passive sentences, but many of them also performed poorly on active sentences. The results of both studies suggest that the underlying deficit that gave rise to the difficulties of the children with ASD in this task may have been different in nature from that of children with DLI.

Durrleman et al. (2016) made a similar observation regarding the across-the-board pattern of deficit in ASD, this time in structures derived by Wh movement. They tested children with ASD aged 6–16 years on the comprehension of Wh questions and relative clauses of various levels of syntactic complexity. They found that the ASD group performed poorer than a group of younger TD children. Importantly, the children with ASD showed difficulty across the board, including in simple sentences, and not only in the sentences with syntactic movement with configurational intervention (in which the full NP object moves across a full NP subject). This, again, indicates that their deficit is different in nature from that of SyDLI children, who typically show differential performance in syntactically simple and in complex sentences, and who show clear effects of configurational intervention (Friedmann et al., 2015).

These syntactic studies thus showed that when ASD is tested with syntactic structures that are clinical markers for syntactic DLI, some children with ASD show impaired performance. However, not all children with ASD show syntactic impairments, and when they do, they sometimes show different error types and different patterns of performance. The next important step forward in our understanding of the relation between ASD and DLI comes from recent studies that compared directly between the performance of these two populations in the syntactic domain, which used specific syntactic structures that may yield differential performance in the two groups, and which looked at error types in the two groups.

Durrleman and Delage (2016) tested the production of pronominal clitics in a group of French-speaking children with ASD and a group of children with DLI. They compared 3rd person accusative clitics, known as a clinical marker for DLI in French (Parisse and Maillart, 2004; Jakubowicz and Tuller, 2008; Tuller et al., 2011), and first person accusative clitics. They found that the ASD and DLI groups performed similarly on third person accusative clitics and in sentence completion testing verbal inflection, prepositions, and passive. The groups differed on first person clitics, which were impaired in ASD (even for children with ASD whose grammar was normal), but mastered by all children with DLI. These results indicate the different sources of impairments in ASD and DLI: third person clitics may require specific syntactic abilities, whereas the use of first person clitics involves pragmatic abilities. Importantly, Prevost et al. (unpublished manuscript) found that once the pragmatic demands on the use of first person clitics are relieved through explicit instructions, children with ASD can actually produce first person clitics similarly to TD children.

Tuller et al. (2017) tested French-speaking children with ASD and children with DLI on sentence-picture matching, sentence repetition, and sentence elicitation tasks, and also analyzed samples of their spontaneous speech. They found that the two groups had similar morphosyntactic performance. The subgroup of ASD who had LI showed impaired performance in three domains that are impaired also in DLI: pronominal clitics, reduced use of embedded sentences, and a large rate of erroneous complex sentences.

Finally, in a recent study, Creemers and Schaeffer (2016) provided another clear and elegant demonstration of the differences between these groups. They compared Dutch-speaking ASD and DLI participants using a lexical-syntactic task of mass-count distinction, and a pragmatic task that tested the use of definite markers. The ASD participants outperformed the DLI participants on the grammatical mass-count task, in which they performed at the TD level, but performed below the DLI when they had to provide a definite determiner, a task that requires pragmatic abilities (Armon-Lotem and Avram, 2005; Balaban et al., 2016; Schaeffer, 2016).

Studies comparing individuals with ASD to individuals with DLI thus focus on syntactic structures that are known to be sensitive markers for syntactic DLI in the relevant languages. In Hebrew, the structures that are most indicative of a syntactic impairment for school-aged children and adults are structures derived by a syntactic movement called “Wh movement” (because this is the type of movement that derives Wh questions), such as relative clauses, topicalized structures, and Wh questions (Friedmann and Novogrodsky, 2004, 2007, 2011; Novogrodsky and Friedmann, 2006; Levy and Friedmann, 2009; Friedmann et al., 2015). These structures with Wh movement are demonstrated in examples 1–3. Relative clauses (example 1), topicalized sentences (2), and Wh questions (3) are all derived by the same type of syntactic movement: movement of a noun phrase to the beginning of the sentence (to spec-CP, in syntactic terms). This movement is schematized by an arrow in examples 1–4, and the position from which the noun phrase has moved (sometimes referred to as “the gap” or the “trace of movement”) is marked by an underline.

Examples for structures derived by Wh movement in Hebrew:

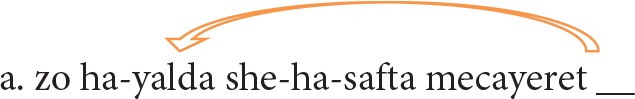
  This the-girl that-the-grandmother draws
  This is the girl that the grandmother is drawing __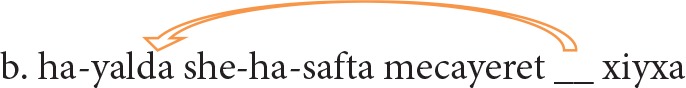  The-girl that-the-grandmother draws smiled
  The girl that the grandmother is drawing __ smiled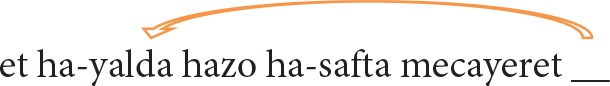  ACC the-girl the-this the-grandmother draws
  This girl, the grandmother is drawing __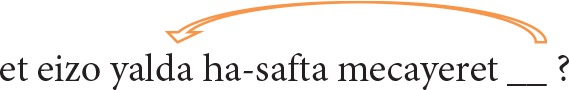  ACC which girl the-grandmother draws?
  Which girl is the grandmother drawing __?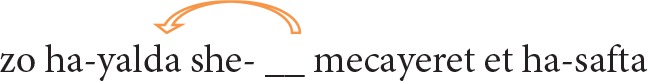  This the-girl that-draws ACC the-grandmother
  This is the girl that __is drawing the grandmother

Relative clauses can be created by movement of the subject NP (as in Example 4) or of the object NP (example 1). Whereas both types of relative clause are derived by Wh-movement, object relatives have been shown to be more impaired than subject relatives in Hebrew SyDLI (Friedmann and Novogrodsky, 2004, 2007, 2011; Novogrodsky and Friedmann, 2006; Levy and Friedmann, 2009; Friedmann et al., 2015). This difference has been ascribed to the different properties of the movement in the two structures: whereas in subject relatives the movement does not change the canonical order of the agent and the theme, in object relatives the object noun phrase moves across the subject noun phrase, and this movement is problematic in SyDLI (Friedmann et al., 2009, 2015; Hamann and Tuller, 2015)^2^.

These structures in which the (full noun phrase) object undergoes Wh movement across another full noun phrase are acquired around age 6 in Hebrew-speaking TD children (Varlokosta and Armon-Lotem, 1998; Günzberg-Kerbel et al., 2008; Friedmann et al., 2009). (The sentences with object relatives in 1a and 1b differ with respect to the position of the relative clause within the sentence—in 1a it is in the end of the sentence, and it is therefore called a “final branching” or “right branching” relative clause—this type of object relative is acquired in Hebrew around age 6. Sentence 1b includes a relative clause in the middle of the sentence (“center-embedding” relative clause), between the subject and the main verb. This kind of relative clause is acquired in Hebrew around 4th grade, age 9–10).

In the current study, we assessed the comprehension and production of these structures in ASD and SyDLI to study in detail the similarities and differences between the two groups. We analyzed the individual performance of each participant in order to examine the degree of heterogeneity within the group. To examine our research question, whether the language difficulty in ASD can be characterized as SyDLI, we looked at the patterns of performance of each participant across different sentence structures, to see whether the ASD participants show a differential pattern that resembles that of SyDLI, and analyzed response types in detail, to examine the differences in error types between the two groups. If the language difficulty in ASD is similar to SyDLI, we would expect to see similarity in the patterns of performance—the ASD participants should show the same distinctions between impaired and intact structures as the SyDLI participants. We would also expect the ASD participants to make the same types of errors as children with SyDLI.

## Methods

### Participants

#### ASD participants

Participants in the ASD group were 18 Hebrew-speaking children and adolescents with autism (16 boys) aged 9;0–18;0 years (*M* = 13;4, SD = 3;1. Nine of the participants were in 9th−11th grade, and nine were in 3rd−5th grade); all were taking part in a larger study of language skills in ASD at Tel Aviv University. Hebrew was the native language for all participants. All the participants were diagnosed with Autism Spectrum Disorder by a child psychiatrist prior to the study according to the DSM-IV criteria (American Psychiatric Association, 2000), and were recommended for an ASD-specific class^3^. Seventeen of the participants with autism were enrolled in autism-specific classes, and the remaining child received 1:1 support in a mainstream class. Thirteen were diagnosed as “High functioning”, indicating that standard psychological assessment found their IQ to be normal. (The other 5 were diagnosed as “PDD-NOS”- Pervasive developmental disorder not otherwise specified—which bears no information as to their IQ). No participant was diagnosed as having “Low functioning” autism, defined as an IQ score <75. Appendix A in Supplementary Material includes information on each of the participants: age, gender, and performance in lexical tasks (picture naming, word-picture matching), and in nonword reading. It also includes scores in a nonverbal task of picture association testing conceptual relations and world knowledge, which can be used as a proxy for nonverbal IQ.

#### Syntactic DLI participants

The participants in the syntactic DLI (SyDLI) groups in this study all participated in previous studies of syntactic DLI in our lab. Each of them was extensively tested for syntax, lexical retrieval, and phonological abilities, and each of them was found to be syntactically impaired. We took their raw data and re-analyzed the measures we selected for this study that would allow us to compare them to the ASD participants.

The SyDLI group to which we compared the performance of the ASD participants in the first task, the elicitation of subject and object relative clauses with pictures, was taken from the children tested by Novogrodsky and Friedmann (2006). It included 16 Hebrew-speaking children with SyDLI (12 boys), aged 9;3–14;6 (mean age 12;6 years, in 4th–8th grade).

The children with SyDLI to whom we compared the performance of the ASD participants in the second task, the reading and paraphrasing task, were the DLI participants reported in Friedmann and Novogrodsky (2007). These were 15 Hebrew-speaking children with SyDLI (11 boys), aged 9;3–14;5 (mean age 11;6 years, in 4th−8th grade).

The SyDLI comparison group in the third task, the sentence repetition task, were 62 children with SyDLI aged 8;8–9;5 years (mean = 8;4, SD = 3.4) from Fattal et al. (2013).

All participants in these SyDLI groups met the exclusionary criteria for DLI (formerly referred to as “SLI”) as formulated by Leonard (2014): They had no hearing impairment and no recent episodes of Otitis Media, no abnormalities of oral structure or problems in oral function; they showed no evidence of obvious neurological impairment; they had no symptoms of impaired reciprocal social interaction or restriction of activities that are typical of ASD. All of the DLI participants had normal IQ and were attending regular classes in regular schools.

#### Typically-developing control participants

The typically-developing (TD) control group for Experiment 1 included 15 TD children aged 9–10 years (mean = 9;2, SD = 2;2). These children were age-matched to the youngest participants in the ASD group. All were studying in 4th grade in regular classes.

The TD control group for Experiment 2 included 61 children aged 9;0–18;1 (mean age = 10;5, SD = 2.5).

The control group for Experiment 3 were 90 TD children aged 5;2–18 years (*M* = 8;9, SD = 4.02). This group was comprised of 40 younger TD children aged 5;2–6;9 years (*M* = 5;8, SD = 0.3) from Fattal et al. (2011) and Friedmann et al. (2010). These children were on average 3 years younger than the youngest children in the ASD group, and at a chronological age at which Hebrew-speaking children have already (just) acquired relative clauses and Wh questions according to previous research (Friedmann and Novogrodsky, 2004; Friedmann et al., 2009, 2015; Fattal et al., 2011; Szterman and Friedmann, 2015). The older group included 50 TD children aged 9–18 years (*M* = 11;6, SD = 2;6). We compared the younger and older participants' performance on each of the 5 sentence types, and none of these sentences types showed a difference between the groups (all *p*'s > 0.32). The analysis of the total correct performance in the two groups also yielded no significant difference [*t*_(88)_ = 0.55, *p* = 0.30]. Therefore, we lumped the results of all the 90 TD children together and treated them as one control group.

All the children in these three TD control groups had no reports of hearing loss, neurological development difficulties or socio-emotional problems. They were studying in public schools serving a middle class population, similarly to the participants with ASD and SyDLI.

The selection of the two comparison groups for the ASD group—the DLI and the TD groups—was done on the basis of the following rationale: we tested syntactic structures that are all already mastered by Hebrew-speaking children at age 9 (fourth grade) (the structures tested in Experiments 1 and 3 are acquired by age 6, the structure tested in Experiment 2 is acquired in 4th grade, around age 9). TD children have high scores in the 3 tasks we used in the current study by age 9, and then performance reaches plateau, so there is no change in scores after this age. We, therefore, selected ASD participants only from age 9 years and up, and compared their performance to children at ages that are supposed to already master these structures. This age-matching can be taken as “Wh-movement-age-matching”—TD individuals aged 9 and above are performing similarly in the tasks we used, so they may all be considered as being of the same Wh movement-mastery age^4^.

As we will show below, the results of our study undermine the validity of matching by language test score: one could say that the ASD participants were matched to the SyDLI participants by syntactic test scores: as we report below, their total scores in the three tests did not differ from that of the SyDLI group. However, we found critical differences between the groups with respect to the types of the errors they made and the patterns of impairment and the sparing of the various syntactic structures, indicating that a similar test score does not indicate that the abilities are similar.

Additionally, the matching by some measure that is not age (e.g., IQ, vocabulary, lexical retrieval) which was applied in some earlier ASD studies was probably based on the assumption that this measure correlates with the ability tested. We found no correlations in the current study between any of the measures presented in Appendix A (Supplementary Material)—nonverbal conceptual ability, lexical retrieval/vocabulary, or reading decoding ability—and the ASD participants' performance in any of the syntactic tasks (tested with Pearson correlation and Bonferroni correction). So matching to control group by these measures is not warranted.

### Tasks

#### Experiment 1: production of subject and object relative clauses

**Figure 1:**
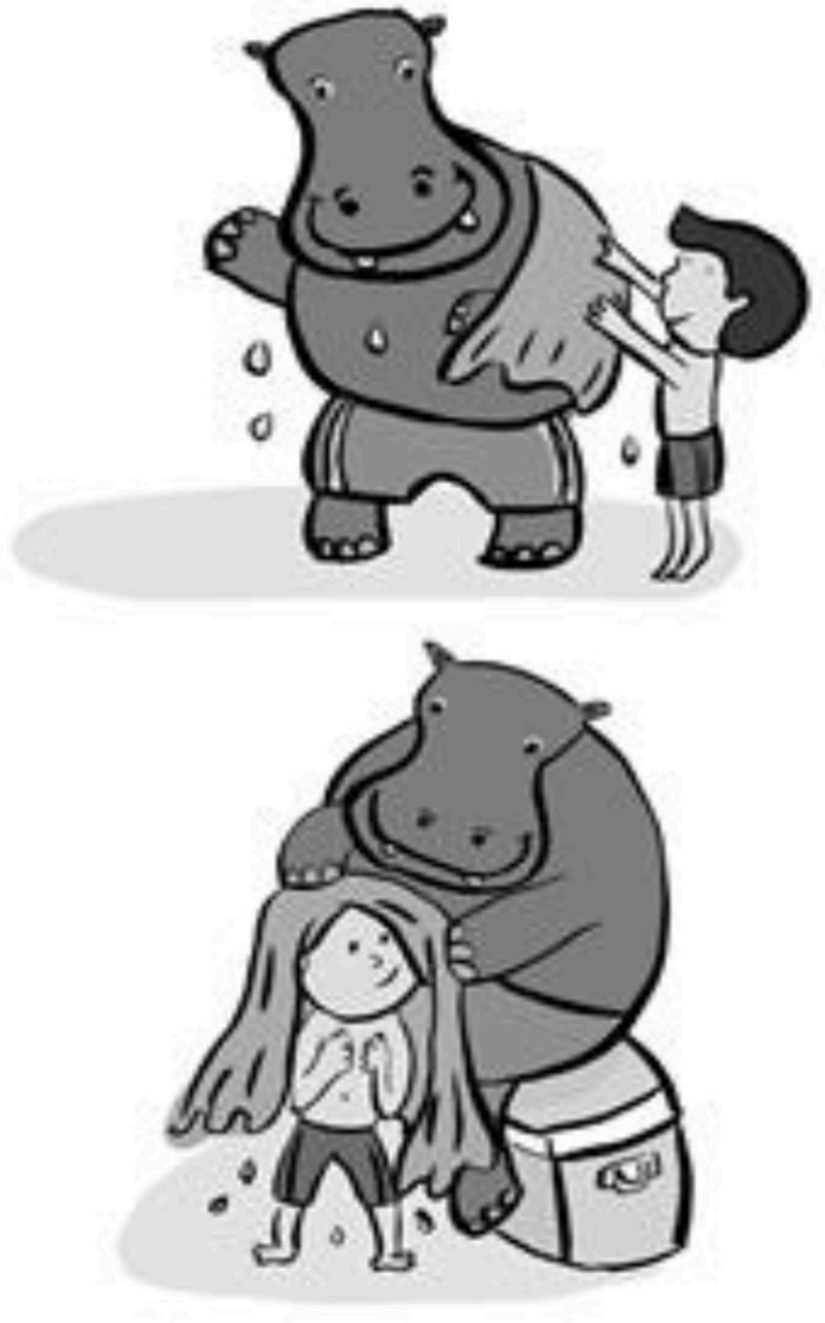
An example of a picture pair presented in the relative clause elicitation task (Experiment 1).

We tested the participants' production of subject and object relative clauses using a sentence elicitation task with pictures (BAFLA ZIBUV test, Friedmann, 1998). The participant was shown a page with two pictures. Each of the two pictures on the page included the same two figures. In the top picture, one figure was performing an action on the second figure and in the bottom picture the roles were reversed. The experimenter described the two pictures using simple sentences and then asked the participant about one of the figures in each picture. The participants saw 10 picture pairs, and were asked one question about one figure in each picture (see Figure 1 and Example 5). One question was targeted at producing a subject relative and one at an object relative, with a total of 10 target subject relatives and 10 target object relatives. The order of the subject and object relative target sentences was randomized across the picture pairs.

(5) The experimenter presented the pictures in Figure 1 and said: “There are two boys in these pictures. In one picture the boy is drying the hippo, and in one picture the hippo is drying the boy. Which boy is this? (pointing to the boy in the top picture) …and which boy is this? (pointing to the bottom one, after the participant provided an answer to the first question). Start your answer with “This is …”.”
  - **Target subject relative**: describing the boy in the top picture in Figure 1.
    ze ha-yeled she-menagev et ha-hipo
      This is the boy that is drying the hippo.
  - **Target object relative**: describing the boy in the bottom picture in Figure 1.
    ze ha-yeled she-ha-hipo menagev
      This is the boy that the hippo is drying.

Before the beginning of the task, an example question was shown to each participant to make sure the participant understood the task and the requirement for starting with “This is …” was introduced. This practice item was not included in the data analysis. If it seemed that the participant did not fully understand the task, the experimenter demonstrated the requested response to the practice item and asked the participant to do as she did (for details about this task see Friedmann and Szterman, 2006; Novogrodsky and Friedmann, 2006).

In the analysis of this test's results, we counted the number of correctly produced target object relatives and subject relatives in comparison to the SyDLI and the TD children. Error analysis was done based on the error analysis described in Friedmann and Novogrodsky (2007) and new error categories were added according to new error types that appeared in the current study (mainly in the ASD group).

#### Experiment 2: reading and paraphrasing of object relatives with heterophonic homographs

This task tested the participants' ability to understand and paraphrase written relative clauses and their ability to correctly read a heterophonic homograph whose correct reading aloud critically hinges upon the correct parsing of the grammatical structure of the sentence (BAFLA ZIKRIA, Friedmann and Gvion, 2003; Friedmann and Novogrodsky, 2007).

The task included ten verb-noun heterophonic homographs, each of which appeared in two sentences—once in a sentence with a relative clause, and once in a similar, length-matched, simple sentence. The homograph was the main verb in all the sentences. The relative clauses were center-embedded object relatives in which the heterophonic homograph appeared right after the trace (which is the original position of the moved object, right after the embedded verb, marked by an underline in Example 6).

Example (6) is an object relative clause with the homograph “gazar”, the verb *cut*. This homograph can be read in other sentence contexts as the noun “gezer”, *carrot*. Example (7) is a simple sentence with the same homograph.

(6) Ha-baxur she-ha-yeled ahav *** __ gazar*** itonim yeshanim.
  The-guy that-the-boy loved cut-past newspapers old
  *The guy that the boy loved was cutting old newspapers*.
(7) Ha-yeled mi-kita daled ***gazar*** itonei sport.
  The boy from-class fourth-grade cut-past newspapers-of sport
  *The boy from fourth grade was cutting sports magazines*.

The sentences were split into two blocks of 10 sentences, each block containing 5 object relatives and 5 simple sentences in random order. Each block was administered in a separate session. Each homograph appeared only once in each block; in one block it appeared in a relative clause and in the other block—in a simple sentence.

The participants were asked to read the sentence out loud and then explain it in their own words. If it seemed that the participant was unable to explain the sentence, s/he was asked a leading question (e.g., “*Who cut*?” For details on this task, see Novogrodsky and Friedmann, 2006; Szterman and Friedmann, 2014).

In the analysis of this test's results, we scored separately correct responses for reading the homograph and for paraphrasing of the sentence, and compared relative clauses and simple control sentences. Three of the ASD participants had difficulties reading the homographs because of their inaccurate reading of the rest of the sentence (they also demonstrated considerable difficulties in testing reading at the single word level) and expressed considerable frustration with the need to read in this task, so the sentence was read to them by the experimenter, and they were only asked to explain the sentence. These participants' results are reported only for the paraphrasing part.

We coded a paraphrase as correct if the participant identified correctly the agent and theme of the two verbs in the sentence.

#### Experiment 3: sentence repetition task

The sentence repetition task is a way of testing the participant's ability to process grammatically complex structures, in a simple task, which allows the comparison between sentences of various structures using the same task (Friedmann and Lavi, 2006; Szterman and Friedmann, 2015). In this sentence repetition task (PETEL, Friedmann, 2000), the experimenter said a sentence, and the participant was requested to count to three out loud and then repeat the sentence, as accurately as possible. The counting was included to prevent phonological memorization in the phonological loop (Baddeley, 1997; Friedmann and Grodzinsky, 1997; Szterman and Friedmann, 2015).

We used this sentence repetition task because previous studies indicated that it is a task that is very sensitive to syntactic impairment in SyDLI, agrammatic aphasia, and in children who are still in the process of acquiring syntax (Lust et al., 1996; Friedmann and Grodzinsky, 1997; Friedmann, 2001, 2007; Friedmann and Lavi, 2006; Fattal et al., 2011). The sentence repetition task uses the fact that sentence repetition cannot be a simple phonological reiteration of the input string, but it rather involves understanding the sentence and reproducing it. Consequently, syntactic impairment that affects the comprehension and production of a certain syntactic construct will result in impaired repetition of sentences with this construct.

The test assessed the participants' ability to repeat sentences derived by Wh movement: we tested the repetition of object relatives, topicalization structures, and Wh questions (object and subject questions, see Example 8). We compared these sentences to simple sentences. The simple sentences were included as minimal pairs with the sentences with Wh movement—they were identical to the Wh movement sentences in words and length and only differed from them in that they included no Wh movement. The rationale was that if the participant fails to repeat the sentences with syntactic movement but succeeds on the sentences without movement, this would point to a syntax-specific deficit in Wh movement.

The sentences derived by Wh movement were also compared to two additional types of syntactic movement: movement of the object to subject position, which is a more local movement than the Wh movement (termed “A-movement” or “Argument movement”). This short movement is often tested with passive structures. We tested it in a structure that is far more common in Hebrew—sentences in the order subject-verb in which the verb is an unaccusative verb (Example 10). (The argument of unaccusative verbs is base-generated in the object position, after the verb, so when it appears before the verb, in subject position, it appears there after moving from the original object position). Such structures are already produced by children younger than 2 years old in Hebrew (Friedmann, 2007; Friedmann and Costa, 2011; Costa and Friedmann, 2012; Reznick and Friedmann, 2017), and are far more natural than passive sentences (e.g., in an analysis of spontaneous speech of 61 Hebrew-speaking children aged 1;6–6;1, which encompassed 27,696 utterances, only a single verbal passive was produced, and even this one was ungrammatical, Reznick and Friedmann, 2017. See also Berman, 1997; Jisa et al., 2002, for the scarcity of passives in Hebrew).

We also compared sentences with Wh movement to another type of movement, in which the verb moves to the second position in the sentence (in this movement, the verb moves to a position before the subject, to the C node, and therefore this movement is sometimes termed “V-to-C movement”, see Example 9). Such movement is optional in Hebrew, so the same sentence can appear either with or without the movement of the verb (compared to the simple sentence in Example 12). Finally, we examined the repetition of sentences without any of these movement types but with a different kind of syntactic complexity: embedding, which we examined through sentences with sentential complements of verbs (Example 11).

The test included 70 sentences: 10 object relatives and 10 object topicalization sentences, 5 subject and 5 object *Which* questions, 10 sentences with verb movement to the second sentential position, 10 sentences with A-movement in which the subject appeared before the unaccusative verb, 10 sentences with embedded sentential complement, and 10 simple sentences without Wh movement.

All sentences contained four words (accusative markers, embedding markers, and prepositions were counted with the word to which they cliticize). All the sentences derived by Wh movement were semantically reversible. The simple sentences and the sentences with verb movement to second position included half transitive and half intransitive verbs, and the sentences with embedded clauses included an embedded intransitive verb. The test started with a practice sentence that the participant was requested to repeat, which was used to make sure the participant understood the task. This sentence was not part of our data analysis.

If the participant was unable to count to three and then repeat the sentence for five sequential sentences, s/he was asked to repeat the sentence immediately without counting. (Three ASD participants could not count before repeating). Sentence types included:

**(8) Sentences with Wh movement**
  - Object relative
    zo ha-talmida she-ha-mora ohevet.
    this (is) the-pupil that-the-teacher likes
  - Topicalization
    et ha-mora ha-zo ha-talmida ohevet.
    ACC the-teacher the-this, the-pupil likes
  - Object Wh question
    Et eize mora ha-yalda ohevet?
    ACC which teacher the-girl likes?
  - Subject Wh question
    eizo mora ohevet et ha-talmida?
    which teacher likes ACC the-pupil?
**(9) Sentences with verb movement to second position**
  Etmol biker ha-yeled xaver
  Yesterday visited the-boy a-friend
**(10) Sentences with A-movement**
  Etmol ha-yeled nafal b-a-gina
  Yesterday the-boy fell in-the-garden
**(11) Embedded sentences**
  Ima amra she-ha-marak nigmar
  Mom said that-the-soup finished
**(12) Simple sentences**
  Etmol ha-yeled biker xaver
  Yesterday the-boy visited a-friend

We analyzed performance in the sentence repetition task by counting for each participant the number of correctly repeated sentences for each sentence type, and compared this to the SyDLI and the TD children. We classified the repetition errors into structural errors and lexical errors. Structural errors are errors that change the thematic grid or the syntactic structure of the sentence. Lexical errors are errors of omission or substitution of a word in the sentence without affecting the thematic roles or the syntactic structure of the sentence. The ASD group made unique errors that did not fit into these error types, and we, therefore, added error categories.

### General procedure

The three tasks reported here were administered to the ASD participants as part of a larger study of language in children with autism. In order to familiarize the participants with the experimenter, she met all the participants in their classrooms 1 day prior to testing sessions for a fun activity. Each child was tested individually in a quiet and familiar room. All children were told they were helping the researcher with a science project and were shown the tape recorder that was recording the session. They were told that they could stop whenever they wanted to go back to class or if they got tired. On completion of each task the children received a sticker and on completion of each session they received a small snack. The number of sessions for each child varied from 2 to 6 sessions, a smaller number of sessions meant longer session duration (an hour on average), whereas a larger number of sessions included shorter sessions (on average 20 min). All sessions were recorded and transcribed. All sessions were held during the morning hours to prevent results being affected by fatigue. Tasks were presented in mixed order across participants. This research was approved by the ethics committee of Tel Aviv University, as well as by the Chief Scientist of the Ministry of Education. The parents of each of the participants signed a consent form informing them of the research aim and nature of the tasks.

### Analyses

For each of the tasks, we analyzed the rate of correctly produced/understood/read/repeated target sentences of each type, and compared the performance in each sentence type to that of children with syntactic DLI and to TD children, using two preplanned comparisons. Because we could not assume normal distribution for the ASD and DLI groups, we compared the groups using non-parametric Mann-Whitney test (which we report with the statistic U, to which we add in parentheses the total N in the two compared groups). A comparison between two conditions within the ASD group was done using the Wilcoxon's signed-ranks test (which we report with the statistic T).

At the individual level, the performance of each participant with ASD was compared with the TD control group using Crawford and Howell's (1998) *t*-test. We used an alpha level of *p* <0.05. This analysis allowed us to examine how many ASD participants performed below the norm for their age in the various tests, and also allowed us to examine the difference between different structures: individuals with SyDLI show difficulties in specific sentence structures, and succeed in other structures. We thus examined, for each individual with ASD, using the Crawford and Howell's (1998) *t*-test, whether their performance was below the control group in all structures or in specific structures^5^.

For each task, we then analyzed the error pattern of each group and compared the distribution of error types in the ASD group to that of the children with SyDLI.

## Results

Below, we report for each task the percentage correct in the ASD group in comparison to that of children with SyDLI and to the TD control group. We then proceed to analyze the types of errors that the participants with ASD made, in comparison to the errors produced by the participants with SyDLI. The results were consistent across the three tasks: Whereas the total percentage correct was roughly the same for the participants with ASD and those with SyDLI, error analysis yielded different error types and different error patterns in the two groups and hence indicated that their deficits were actually different in nature.

### Experiment 1: production of subject and object relative clauses

#### ASD and SyDLI show similar percentage correct production of subject and object relatives

As a group, the participants with ASD performed poorer than the controls on both subject and object relatives [*U*_(33)_ = 49.5, *p* = 0.0006; *U*_(33)_ = 46, *p* = 0.0009, respectively], and similarly to children with SyDLI, *U*_(34)_ = 123.5, *p* = 0.34; *U*_(34)_ = 173, *p* = 0.53, for subject- and object relatives, respectively.

**Figure 2:**
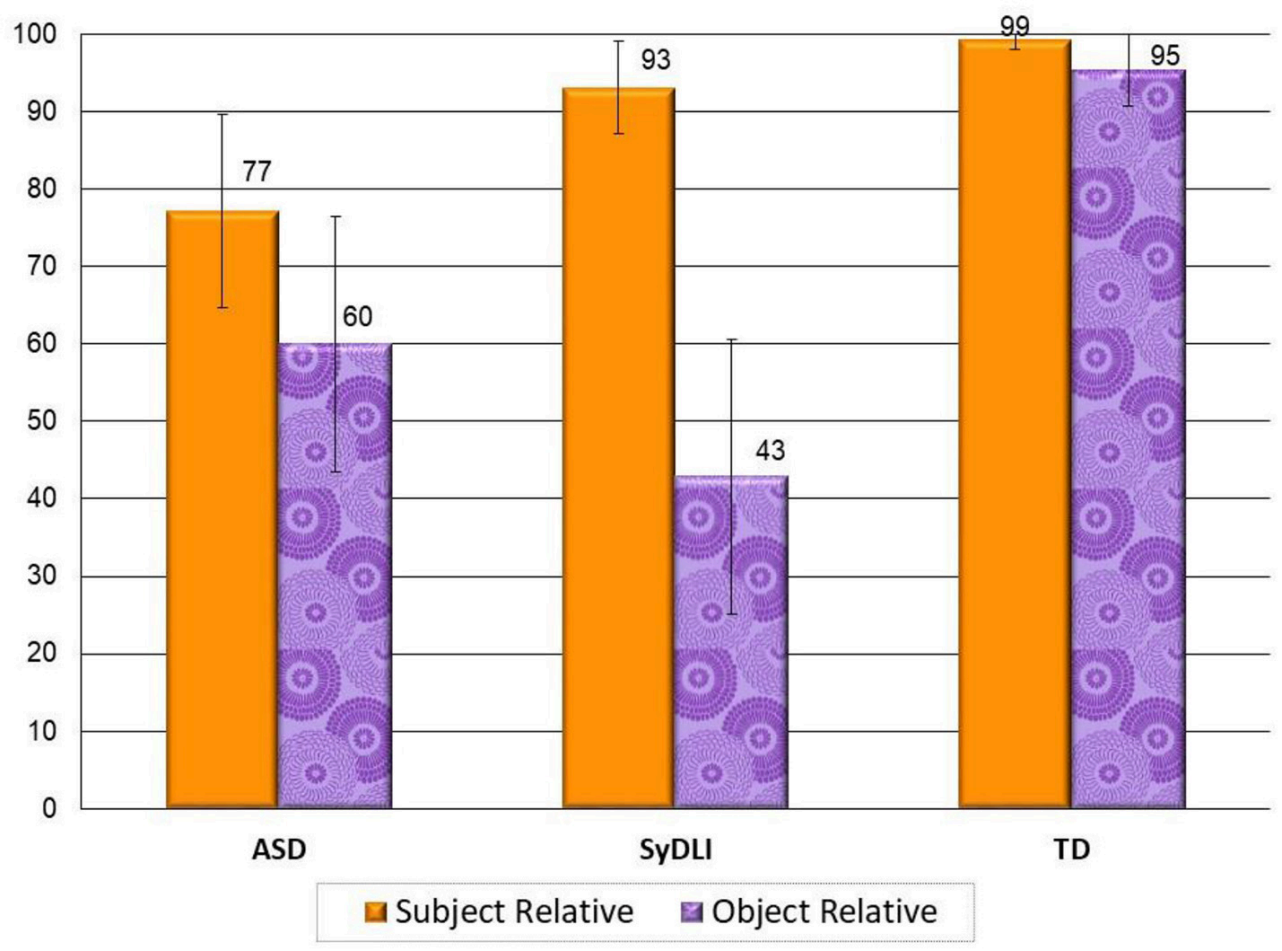
Production of subject- and object relatives in the ASD, SyDLI, and control groups (average % correct, error bars indicate standard deviations).

The percentages of correctly produced subject and object relatives in the three groups are summarized in Figure 2. The participants in the control group produced both subject and object relatives effortlessly and correctly (subject relatives 99% and object relative 95% correct, in line with many previous reports indicating that by the age of 7 years Hebrew-speaking children already master the production of subject and object relative clauses (Friedmann and Novogrodsky, 2004; Friedmann and Szterman, 2006; Novogrodsky and Friedmann, 2006; Friedmann and Costa, 2010; Fattal et al., 2013; Friedmann et al., 2015).

#### ASD and SyDLI show different patterns of performance with respect to the subject-object asymmetry

Once we looked at the pattern of subject vs. object relatives, for each participant, the large difference between the ASD and SyDLI groups started to unfold: The SyDLI group showed more consistent performance within the group and more consistent advantage for subject relatives over object relatives: except for two children with SyDLI who performed at ceiling on both relative clause types, all SyDLI participants produced more target subject than object relative clauses (we included in the target relative clauses also relatives with resumptive pronouns at the gap position, and excluded avoidance of crossing movement).

In the ASD group, the pattern was markedly different. Although as a group their production of object relatives (*M* = 60%) was poorer than their production of subject relatives (*M* = 77%), it does not seem justified to analyze their performance at the group level, as the variance within the ASD group was very large (reflected in the large standard deviations: 33% for the object relatives and 25% for the subject relatives, SDs that were 10 times larger than in the control group).

When we compare the production of object relatives to the production of subject relatives at the individual level in the ASD group, 4 of the ASD participants produced both types of relative clauses like the controls. The other 14 participants performed significantly below the control group (who were younger in age than most ASD participants) on subject relatives, on object relatives, or on both. Of the ASD participants who performed significantly below the control group, 9 participants showed impairment on both subject relatives and object relatives, and 3 ASD participants produced object relatives normally but showed impaired production of subject relatives. Only 2 ASD participants showed a pattern that is similar to that of the children with SyDLI, of impaired production of object relatives, and normal production of subject relatives.

#### ASD and SyDLI show different error types

The ASD and SyDLI groups also markedly differed with respect to the types of errors they committed in this task.

##### Errors in target subject relatives

The types of non-target responses that the ASD and SyDLI (as well as the control) groups produced for the target subject relatives are presented in Table 1. The error pattern of the ASD group crucially differed from that of the SyDLI group in that many of their errors were pragmatic (13% of all their responses, and 57% of their erroneous responses), whereas the SyDLI participants produced only syntactic errors, and no pragmatic errors. An error was considered pragmatic when it was unrelated to the target sentence, the question asked, or the picture presented (see Examples in 13). In the coding of responses, each response was coded separately for grammaticality (syntactically correct or including a syntactic error, or a different syntactic structure than the required one) and for pragmatic felicity (felicitous or with a pragmatic error). Thus, some of the responses were syntactically correct but pragmatically infelicitous, some were pragmatically felicitous but syntactically incorrect, and some non-target responses were both syntactically incorrect and pragmatically infelicitous (and some included more than one type of syntactic error).

**Table 1:** Distribution of responses when a subject relative was expected in the picture description task (% of responses).

**Type of response**	**ASD**	**SyDLI**	**Control**
**Syntactically and pragmatically appropriate subject relative**	77	84	98
**Syntactic Errors**			
Resumptive pronoun at the embedded	5	6	2
subject position			
Doubling of the relative head: full DP at	1	5	0
the embedded subject position			
Simple sentence	5	3	0
Object omission	5	1	0
Other ungrammatical	12	1	0
Role reversal in a correct structure	3	0	0
Arguments that do not match the verb's	2	0	0
argument structure (and morphology)			
**Pragmatically infelicitous responses**	13	0	0

The infelicitous responses sometimes described correctly some aspect of the picture, but, importantly, they were infelicitous with respect to the task and the question the experimenter posed. The TD and SLI participants had no trouble identifying the experimenter's intent, even if they had a problem phrasing their response correctly as a relative clause. The participants with ASD often failed to understand exactly what was expected from them in the task (i.e., select a response that relates to the two options suggested to them in the lead in sentence and the action described in it), and the result were these responses, which were correct picture descriptions, yet infelicitous for the task.

**(13) Examples for pragmatically infelicitous responses in the ASD group**.
  - Subject relative: There are two women in this picture. In one picture, the woman is drawing the girl, and in one picture the girl is drawing the woman. Which woman is this?
    **Target response**: This is the woman who is drawing the girl.
    **Pragmatic error**: *This is the woman with the slippers*.
  - Subject relative: There are two nurses in this picture. In one picture, the nurse is photographing the girl, and in one picture the girl is photographing the nurse. Which nurse is this?
    **Target response**: This is the nurse who is photographing the girl.
    **Pragmatic error**: *This is the nurse who doesn't photograph another nurse at all*.
  - Object relative: There are two cats this picture. In one picture, the cat is biting the dog, and in one picture the dog is biting the cat. Which cat is this?
    **Target response**: This is the cat that the dog is biting.
    **Pragmatic error**: *This is the cat that doesn't bite cats*.

##### Errors in target object relatives

In the target object relative condition, too, the ASD participants produced different error types from the SyDLI participants: 48% of the erroneous responses of the ASD group included pragmatic errors (22% of all their responses), whereas the SyDLI participants (and the TD) produced none. The rest of the errors in both groups were syntactic errors, producing subject relatives instead of object relatives, and reducing the number of full DPs in the sentence, as shown in Table 2 (see Examples 14 and 15).

**(14) Example for a subject relative instead of a target object relative in which one NP is reduced by the use of a reflexive verb:**
  There are two girls this picture. In one picture, the girl is drying the woman, and in one picture the woman is drying the girl. Which girl is this?
  **Target response**: This is the girl that the woman is drying.
  **Thematic role reduction and role reversal:**
  zo ha-isha she-mitnagevet
  *This-is the-woman that-dries-reflexive*.
**(15) Example for an ungrammatical object relative in a target object relative item:**
  There are two boys this picture. In one picture, the boy is hugging the monkey, and in one picture the monkey is hugging the boy. Which boy is this?
  **Target response**: This is the boy that the monkey is hugging.
  **Ungrammatical response with doubling of the head (filled gap):**
  ze ha-yeled she-ha-kof mexabek et ha-yeled
  *This-is the-boy that-the-monkey hugs ACC the-boy*.

**Table 2:** Distribution of responses when an object relative was expected in the picture description task (% of responses).

**Type of response**		**ASD**	**SyDLI**	**Control**
**Syntactically and pragmatically appropriate object relative**		54	46	94
**Syntactic Errors**			
Subject relative	Subject relative instead of object relative	28	23	2
	Subject relative, theta roles incongruent with verb's argument structure (and morphology)	3	9	0
Thematic role reduction	NP reduction: object relative with empty subject, omission of object NP, or use of reflexive	7	12	4
No relative	Simple sentences	3	4	0
Other ungrammatical	Relative head doubling or relative head omission	12	5	0
**Pragmatically infelicitous responses**		22	0	0

Thus, the ASD group differed from the SyDLI group in the types of errors they produced and in the distribution of their errors: The ASD group produced pragmatic errors, whereas the SyDLI group produced only syntactic errors; moreover, the ASD group failed on both subject relatives and object relatives, unlike the SyDLI group, who failed almost exclusively on object relatives.

#### Experiment 2: object relative reading and paraphrasing

The reading and paraphrasing task had several aims: it tested the way individuals with ASD understand written relative clauses, it tested the way they phrase explanations of such sentences (as well as simple control sentences), and it assessed the reading of heterophonic homographs in these sentences, whose correct reading crucially depends on the correct syntactic analysis of the sentences.

##### Different pattern of performance in homograph reading

The children in the SyDLI group made very few errors in reading the homographs, and half of them did not differ from the controls in homograph reading. However, when they did make a mistake in reading the homograph, it was always in the relative clause condition—they did not make homograph reading errors in the simple sentences. Namely, the SyDLI participants' misreading of the homographs was closely related to their failure to understand object relatives^6^.

**Figure 3:**
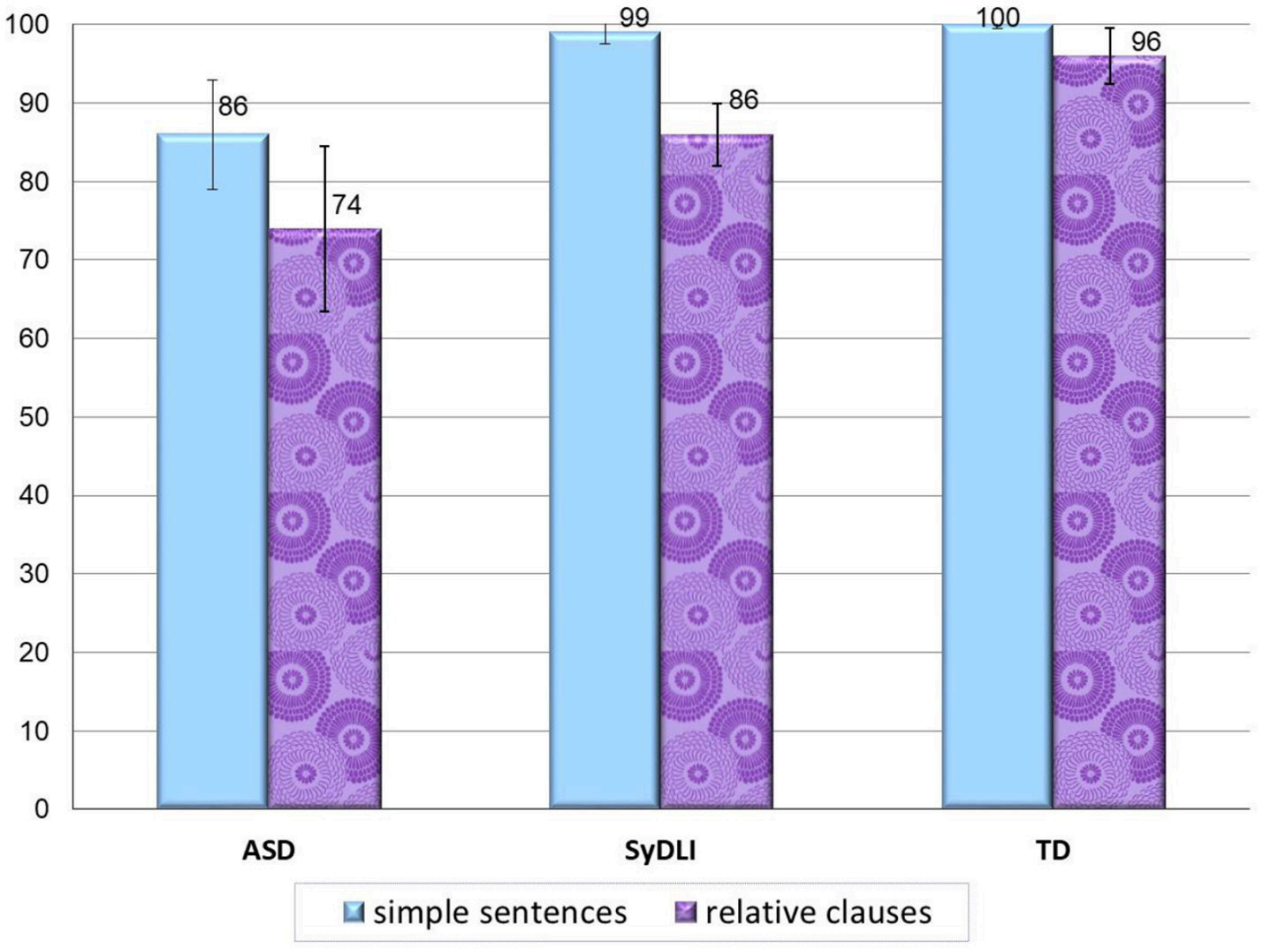
Homograph reading in the relative clauses and simple sentences in the ASD, SyDLI, and control groups (average % correct; error bars indicate standard deviations).

The children in the ASD group showed a dramatically different pattern: of the 15 children with ASD who made reading errors on the homographs, 11 made errors on both the relative clauses and the simple sentences. Namely, they did show difficulty in reading the homographs but, unlike the children with SyDLI, this difficulty was not related to the syntactic structure of the sentence in which the homograph appeared. In the relative clause condition, the ASD group read the homographs significantly poorer than control group [*U*_(79)_ = 922.5, *p* < 0.0001], and similarly to the SyDLI group [*U*_(33)_ = 86, *p* = 0.08]. The important difference was seen in the simple sentence condition, where the ASD group read the homographs significantly poorer than both the SyDLI group [*U*_(33)_ = 57, *p* = 0.002] and the TD group [*U*_(79)_ = 880, *p* < 0.0001]. Figure 3 summarizes the homograph reading in the two sentence types in the three groups. The ASD participants' difficulty with reading the homographs, then, seems not to be related to a syntactic deficit, but rather to the lack of use of information from the semantic system to guide the choice of the correct homograph choice in the phonological output lexicon (see also Brock et al., 2017). It is interesting to note that they did not use the syntactic structrue to guide their choice of the correct pronunciation of the homograph.

##### Different patterns of performance in sentence paraphrasing

**Figure 4:**
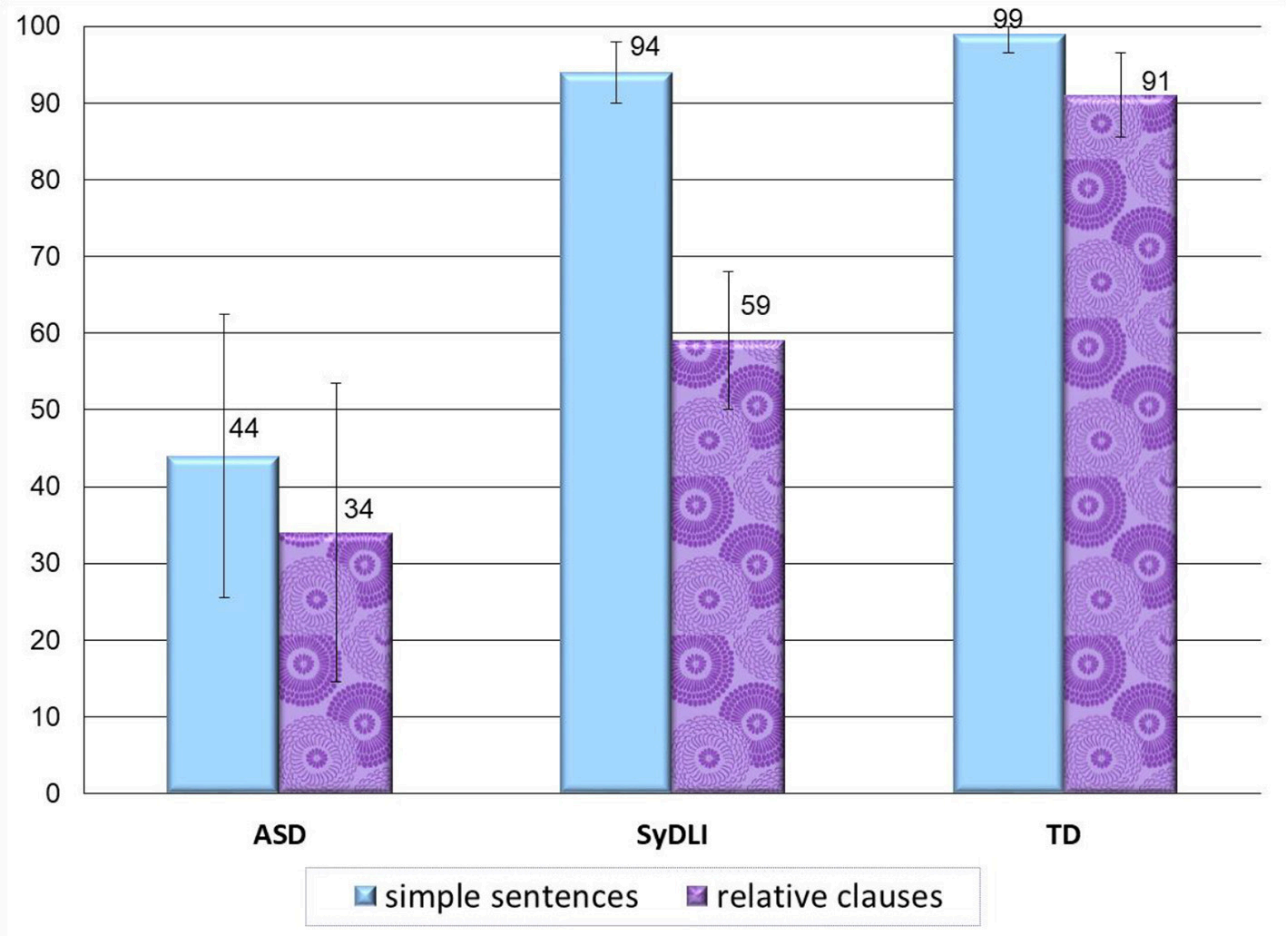
Sentence paraphrasing in the relative clauses and simple sentences in the ASD, SyDLI, and control groups (average % correct; error bars indicate standard deviations).

In the sentence paraphrasing task too, the ASD group showed impaired performance with a different pattern from that of the SyDLI group, as summarized in Figure 4. The main difference was that whereas the SyDLI participants showed significantly better paraphrasing of the simple sentences compared to the relative clauses (*p* < 0.0001), no such difference was found in the ASD group (Wilcoxon's *T* = 30, *p* = 0.17). The ASD participants performed poorly in paraphrasing both the relative clauses and the simple sentences (34 and 44% correct respectively). On the individual level analysis, 14 of the 18 participants performed significantly below the TD group on paraphrasing the object relatives, and 16 participants performed below the TD control participants on paraphrasing the simple sentences.

On paraphrasing the relative clauses, the ASD participants performed below the control participants [*U*_(79)_ = 1022.5, *p* < 0.0001], and did not differ from the SyDLI group [*U*_(33)_ = 96, *p* = 0.17]. Like in the reading analyses, also in paraphrasing, in the simple sentences, the ASD group performed significantly poorer than both the SyDLI group [*U*_(33)_ = 33, *p* = 0.0002] and the control group [*U*_(79)_ = 1022, *p* < 0.0001].

Thus, we see again that the ASD participants, although failing in the paraphrasing task, crucially differed from the SyDLI participants in their error patterns: they failed on both object relatives and simple sentences, whereas the SyDLI participants only failed on paraphrasing the relative clauses.

##### Different error types in sentence paraphrasing

The analysis of the errors that the ASD and SyDLI participants produced when they tried to paraphrase the sentences shed further light on the differences between these groups. The decisive majority of paraphrasing errors of the children with SyDLI were thematic role errors—failing to understand who the agent and the theme in the sentence are. The children with ASD showed a very different pattern: as summarized in Table 3, they almost never made such thematic role errors. In fact, only one participant did so in a single paraphrase. This surprised us, and we checked and re-checked their errors, but indeed they did not. Instead, they provided types of responses we did not see in the paraphrases of the SyDLI and TD groups. They often (24 of their responses) simply provided a word or **several words** from the target sentence, with no specific structure (Examples 16–18). In other cases they provided a response that **did not explain the sentence** or reflected complete failure to understand the sentence (Examples 19, 20). Of these responses, 32 responses were cases in which the ASD participants used a **pronoun** to explain a sentence, even though there was no way for the experimenter to know to which NP this pronoun was referring (Example 21). We found this kind of response especially interesting because the use of pronouns without establishing a reference in discourse is a landmark of the discourse of individuals with Theory of Mind (ToM) impairment (Balaban et al., 2016). In other cases, some ASD participants chose an avoidance strategy of saying “**I don't know**” even when asked guiding questions to see if they understood the sentence. Even genuine attempts by the experimenter to lead the participant to provide an explanation of the sentence often arrived at a dead-end (see Examples 16 and 17).

**Table 3:** Distribution of the various types of paraphrasing errors (% out of all the correctly read sentences).

**Response type**	**ASD**	**SyDLI**	**TD**
Thematic role errors	0.3	34.6	8
Paraphrasing only the main clause	0	6.6	4.9
Providing some words from the sentence in no specific order or structure or an explanation that does not explain the sentence, that uses an obscure pronoun, or that reflects misunderstanding of the social situation described in the sentence	34.7	0	0
“Don't know”	4.5	0	0
Repeating the written sentence exactly or with a random change in the sentence	15.9	4.1	3.9

In addition, all three groups sometimes **repeated** (re-read) the written sentence instead of explaining it, but this type of non-target response occurred more often in the ASD group. When they repeated a sentence instead of explaining it, the ASD participants sometimes randomly changed the inflection of a verb in the sentence they repeated (sometimes from the present to past or vice versa, and once even changing the number agreement of the verb, so that the verb no longer agreed with the rest of the sentence). They sometimes also repeated only part of the sentence in a way that did not yield a sentence (e.g., explaining the sentence *The judge that the man drew speaks on the radio*: “*That the judge that the man drew*”). This did not happen in the two other groups.

**(16) Target:** ha-baxur she-aba cilem megadeal taltalim arukim
  *The guy that daddy photographed grows long curls*.
  **paraphrase**: baxur! (experimenter: ma ha-mishpat omer?) taltalim! (experimenter: le-mi yesh taltalim?) le-aba? Ein po taltalim. Axshav ani mesupar.
  *A guy*! (Exp: what does the sentence say?) *curls*! (exp: who has curls?) *daddy*? *There are no curls here. Now my hair is cut*.
**(17) Target**: ha-xaver she-aba hevi mesarek l-a-yalda et ha-cama
  The friend that daddy brought combs the braid of the girl
  **Paraphrase**: cama! (experimenter: le-mi yesh cama?) l-a-yalda. (experimenter: yesh po od mishehu b-a-mishpat? mi?) ken. Yeled. (ma hu ose?) klum.
  *braid*! (Exp: who has a braid?) *the girl!* (exp: is there anyone else in the sentence? who?) *yes, a boy*. (exp: what does he do?) *nothing*.
**(18) Target**: Ha-leican she-ha-yeled raa metaken sulamot b-a-kirkas
  The clown that the boy saw is fixing ladders at the circus
  **Paraphrase**: Yeled, Leican, Sulam, Kirkas.
  *boy, clown, ladder, circus*.
**(19) Target**: Ha-leican she-ha-yeled raa metaken sulamot b-a-kirkas
  The clown that the boy saw is fixing ladders at the circus
  **Paraphrase**: Kirkas. Atem yod'im ma ze kirkas? Yesh sham harbe cva'im ve-yeladim. ve-yeladot.
  *Circus. You know what a circus is? there are many colors and boys there. And girls*.
**(20) Target**: ha-baxur she-ha-yeled ahav gazar itonim yeshanim
  The guy that the boy loved cut old newspapers
  **Paraphrase**: ha-yeled katav.
  *The boy was writing*.
**(21) Target**: Ha-shofet she-ha-ish ciyer medaber ba-radio
  The judge that the man drew speaks on the radio
  **Paraphrase**: hu mecayer ve-maklit oto l-a-radio.
  *He is drawing and recording him to the radio*.

##### Variability within the ASD group and indications for ability to produce relative clauses in the paraphrases

Another insight into the ASD participants' syntax can be gained from analyzing the syntactic structures of the sentences they produced in their paraphrases. When we look at each of the 18 individuals with ASD, we see that 15 of them used voluntarily and correctly at least one relative clause when they attempted to explain the sentences, and 13 of them even produced an object relative (which is notoriously difficult to produce even when directly elicited in the SyDLI group). Indeed, some of these relative clauses were produced within responses that did not explain the target sentence, but the syntactic machinery constructing relative clauses seems to be working. (For example, to explain *The guy that the boy liked cut newspapers*, one participant said “This is a guy… [exp: what did he do?] …took the boy who liked carrots”, spontaneously producing a subject relative). Three ASD participants did not produce even a single relative clause in their paraphrases.

To conclude, in the sentence paraphrasing task we saw a general pattern that was similar to the one we found in Experiment 1: the ASD participants performed very poorly in this syntactic task, but their patterns differed from that of the SyDLI participants: they made errors on both the relative clauses and the simple sentences, and the types of responses they provided were very different from those provided by the SyDLI participants. The syntactic structures that they used in their explanations actually showed that they are able to use relative clauses (semi) spontaneously.

#### Experiment 3: complex sentence repetition task

##### Different patterns of performance in repetition of the various sentence structures

**Figure 5:**
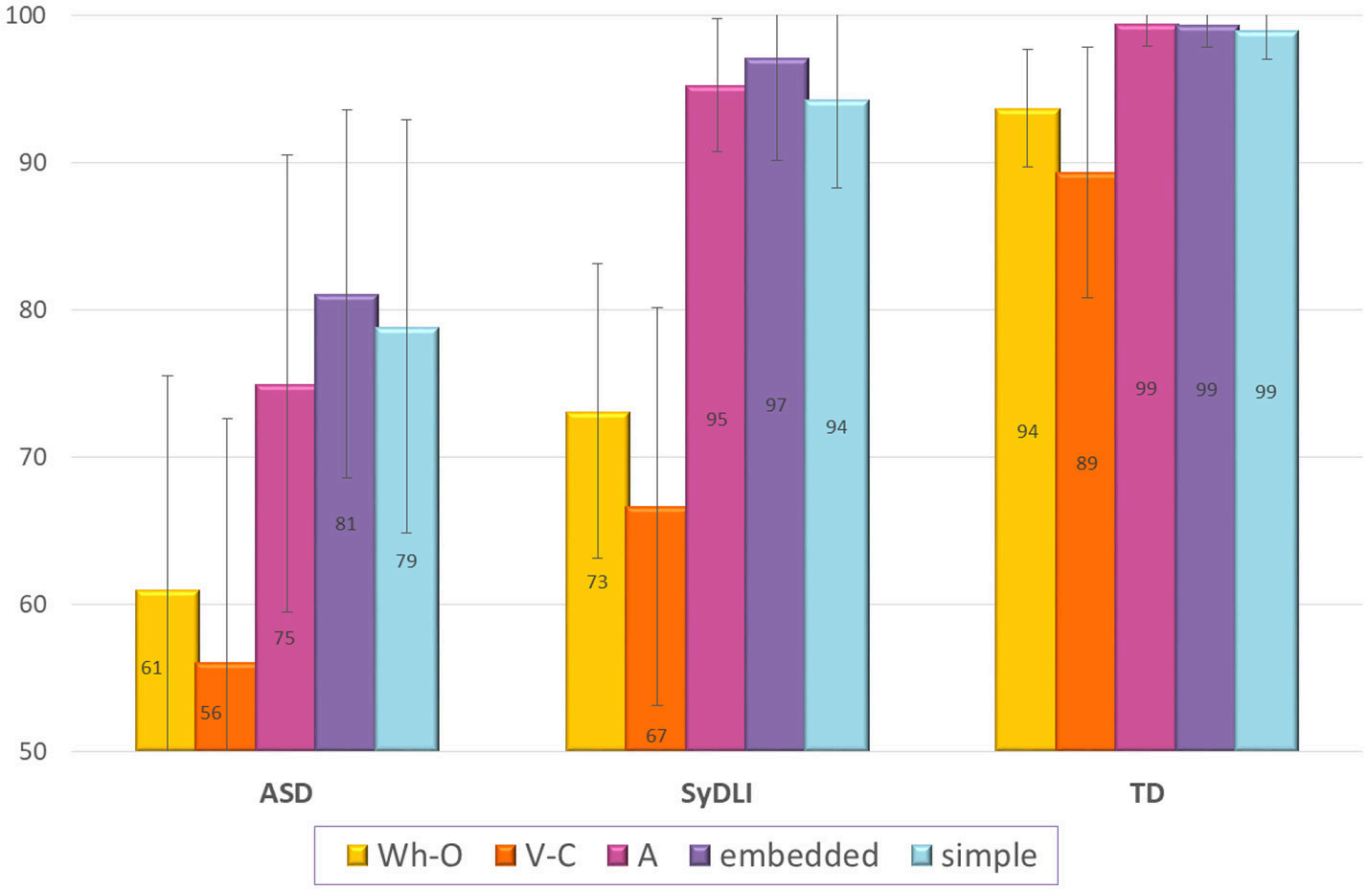
Repetition of the various syntactic structures in the ASD, SyDLI, and control groups (average % correct; error bars indicate standard deviations). Wh-O, sentences with Wh movement of the object: topicalization; object relative, object Wh question; V-C, verb movement to second sentential position. A, A-movement of the theme of an unaccusative verb—from object to subject position; embedded, sentential complements of verbs; simple, SV sentences without any of the above movements and without embedding.

In the sentence repetition task, again, most children with ASD showed a completely different pattern from the one evinced by the children with SyDLI. The participants with SyDLI showed impaired repetition of sentences with Wh movement (and some of them also in sentences with V-to-C movement) alongside much better and above 94% correct in the repetition of embedded sentences, sentences with A-movement, and simple sentences, as shown in Figure 5. The children with ASD did not show this selective syntactically-principled difficulty, and repeated correctly less than 81% of the sentences in each of these 5 structures [their repetition was significantly poorer than that of the controls on each of the sentence types: sentences with Wh movement: *U*_(108)_ = 1,485, *p* < 0.0001; verb movement: *U*_(108)_ = 1,400.5, *p* < 0.0001; A-movement: *U*_(108)_ = 1,393, *p* < 0.0001; Embedded sentential complements: *U*_(108)_ = 1,227.5, *p* < 0.0001; simple control sentences: *U*_(108)_ = 1,310, *p* < 0.0001].

As both the ASD and the SyDLI groups performed poorly on the Wh movement and the verb movement structures, the ASD group did not differ from the SyDLI group on Wh movement [*U*_(80)_ = 730.5, *p* = 0.05, which would be decisively nonsignificant once a correction for multiple comparisons is applied] or verb movement [*U*_(80)_ = 650.5, *p* = 0.30]. However, only the ASD participants failed on the A-movement sentences, the embedded structures, and the simple sentences, so the ASD group performed significantly below the SyDLI group on these structures [*U*_(80)_ = 885, *p* < 0.0001; *U*_(80)_ = 758, *p* = 0.004; *U*_(80)_ = 828.5, *p* = 0.0001, respectively]. In addition, unlike the SyDLI group, the ASD participants showed impaired repetition not only of the object Wh questions, but also of the subject Wh questions (which they repeated only 72% correct), with no significant difference between the two. Namely, one straightforward difference between the ASD and SyDLI performance on the sentence repetition task was that the children with ASD showed impaired repetition of all the tested syntactic structures, including the simple sentences, whereas the SyDLI participants showed a deficit that was specific to Wh movement (and some of them also to verb movement).

##### Large variability within the ASD group

As was the case in the two previous tasks, the variance within the ASD group was very large. When we compare, for each individual with ASD, the performance in the various sentence structures in comparison to the 91 control participants (using Crawford and Howell's, 1998, *t*-test), we find many different patterns of impairment: 5 of the 18 ASD participants had very low scores on all sentence types; 3 participants had very low scores on all sentence types but the simple sentences; 2 had normal repetition of embedded sentences and sentences with Wh movement but showed very impaired repetition of the other sentence types; One participant had high scores on simple sentences and embedded sentences but very low scores on the rest; One participant had very low scores on all sentences excluding embedded sentences where he performed at the control group level; One participant had extremely low scores on verb movement but control level scores on all other sentences. Whereas 14 of the 18 ASD participants had a total score that was significantly below that of the control group, only 4 ASD participants showed the performance that is typical of SyDLI, with poorer-than-normal repetition of sentences with Wh movement and good repetition of the other structures, and one participant showed poorer-than-normal repetition of verb movement and Wh movement, similar to the response pattern of some of the SyDLI participants.

##### Different error types in the ASD and SyDLI groups

As in the previous two experiments, in this task, too, the ASD group differed from the SyDLI group also with respect to the types of errors they produced when they failed to repeat a sentence. We classified the errors into structural and lexical. An error was coded as structural when the participant produced a sentence using the nouns and verbs that appeared in the target sentence but changed the grammatical structure of the target sentence [either by changing its syntactic structure to a simpler structure (Example 22), or by reversing the thematic roles in the sentence]. Errors were coded as lexical errors when the participant produced a sentence that was structurally identical to the target sentence, but substituted or omitted words in the sentence (in most cases to a semantically related word) (Example 23).

**(22) Target**: ze ha-yeled she-ha-shaxen pagash
  This-is the-boy that-the-neighbor met
  **Structural error**: ze ha-yeled she- pagash et ha-shaxen
  This-is the-boy that-met the-neighbor
**(23) Lexical error**: ze ha-yeled she-ha-shoter pagash
  This-is the-boy that-the-policeman met

Whereas the errors of the children with SyDLI (and the few errors that the TD participants made) could be classified into one of these two error types, structural errors and lexical errors, the ASD group made other types of errors that were not witnessed in the DLI and TD groups. Even the youngest children in the TD group, who were 5 years old, did not make such errors [these errors were indeed unique for the ASD group—showing a significant difference between the three groups, *Friedman's test* $\chi_{\left(2\right)}^{2}$ = 8927.2, *p* < 0.0001]. The children with ASD made perseveration errors (Albert and Sandson, 1986; Cohen and Dehaene, 1998), in which the participants' previous response persisted and interfered with their new response (where one or more words from a previous response appeared in the repetition of another sentence, see Example 24).

An additional error that was unique to the ASD group was information addition: some ASD participants were able to repeat the target sentence accurately but then would add information that had not appeared in the target sentence (Example 25). In other cases, they changed the target sentence completely, producing their interpretation of it, even though they were told numerous times to only repeat what they heard (Example 26), or providing their free-associations related to the sentence they had heard.

An additional error type that was found only in the ASD group was answer-instead-of-repetition. When requested to repeat a question, some ASD participants simply answered it, instead of repeating it (Example 27).

**(24) Target**: zot ha-mazkira she-ha-rofe pagash
  This is the secretary that the doctor met
  **Production**: zot ha mazkira she-ha-mora pagsha
  *This is the secretary that the* ***teacher****met* (the word “teacher” appeared several sentences before the target sentence, and the child kept perseverating the word “teacher” in his repetition of several sentences that followed the original teacher-containing sentence).
**(25) Target**: etmol ha-yalda nishka et ha-mora
  Yesterday the girl kissed the teacher
  **Production**: etmol ha-yalda nishka et ha-mora ve-et ha-miflecet
  *Yesterday the girl kissed the teacher* ***and the monster***
**(26) Target**: ha-yeled raa she-ha-shoter kafac
  The boy saw that the officer jumped
  **Production**: ha-shoter ba litfos et ha-ganavim
  ***The officer came to catch the thieves***
**(27) Target**: et eizo mora ha-yalda ohevet?
  Which female-teacher does the girl love?
  **Production**: **Mati**! (the name of one of the class' therapists)

Table 4 summarizes the error types in the three groups (some responses included more than one kind of error, in which cases the response was coded for each type of error it included).

**Table 4:** Distribution of the various error types in the sentence repetition task (% of all repetition responses).

	**ASD**	**SyDLI**	**TD**
Structural errors	22	14.7	1.7
Lexical errors	24	10.1	1.2
ASD-unique errors	12	0	0
**Types of unique errors (% of unique errors)**			
Perseveration	79	0	0
Adding information	6	0	0
Associative remarks	8	0	0
Answering a question	10	0	0

#### Individual syntactic abilities across the three tasks

Another step in our quest for an answer to the question “Do ASD individuals have (syntactic) DLI” was to look at each participant's syntactic ability individually. We saw in the three tasks that the participants make many errors that originate in a pragmatic, rather than a syntactic difficulty. Therefore, we excluded these errors, and cast the question as two separate ones: (a) Do any of the ASD individuals have a syntactic deficit that resembles that of individuals with SyDLI (in addition to a pragmatic difficulty?) and (b) Are there individuals with ASD who have completely normal syntax (in addition to a pragmatic difficulty?).

The analysis of the three tasks on the individual level yielded the following answers to these two questions:

- Only one participant showed a syntactic deficit that consistently resembled that of individuals with SyDLI (once we disregarded his responses that were pragmatically infelicitous), with syntactic errors in producing object relatives in the relative clause elicitation task alongside good production of subject relatives that was similar to that of the control participants; thematic role misunderstanding in the relative clause paraphrasing task; and impaired repetition of Wh movement-derived sentences (object Wh questions, relative clauses, and topicalized sentences). Another ASD participant showed impaired production of object relatives with many syntactic errors and avoidance of Wh movement, and very poor repetition of sentences with Wh movement, but his reading of the homographs in the sentences was so poor that it did not allow us to determine whether he understood the syntactic structure and thematic role grid of the sentences he read or not.
- Seven of the ASD participants showed normal syntactic abilities. They produced both subject and object relatives (even though many of their responses were pragmatically odd), understood well the object relatives they read, at the level of the control participants (although their explanations were not always taking the hearer state of knowledge, or the experimenter's requests into account); and did not make syntactic errors in repeating sentences derived by Wh movement (even if they provided their own interpretation to the sentences from time to time or produced perseverations of lexical items from previous sentences)^7^.

This analysis shows the great variability with respect to syntax in the ASD group: seven showed normal syntax in sentences derived by syntactic movement; many participants showed poor performance across various syntactic structures including simple ones, a pattern that differs from that of syntactic DLI; and only one ASD participant showed a pattern that resembles the specific pattern that characterizes syntactic DLI.

## Discussion

A question that arises often with respect to the language abilities of children with ASD is whether they have DLI (e.g., Bishop, 2001, 2003, 2006; Kjelgaard and Tager-Flusberg, 2001; Tager-Flusberg, 2006). This study has a very clear answer for this question: No. We used three tasks of comprehension and production of syntactically complex sentences and compared the performance of children with ASD to children with syntactic DLI. The results of the three tasks were the same: whereas, *prima facie*, the overall correct performance on the syntactic tasks is similar for the children with ASD and those with SyDLI, once we look closer, they are very different. The types of errors that the ASD group makes differ from those of the DLI group. They also differ in the pattern of performance—the children with SyDLI show impaired performance in specific sentence types, and better performance on other types, in a syntactically principled way; the ASD participants show generalized low performance on the various sentence tasks. Additionally, the huge variability within the ASD group was manifested in the analysis at the individual level: some children with ASD had completely normal syntactic performance, some showed difficulties in the syntactic tasks but these looked very different from that of children with SyDLI, and only one participant showed the syntactic pattern shown in SyDLI.

This general pattern can be seen in each of the three tasks in this study: Experiment 1 examined the production of subject and object relative clauses, a domain that has been repeatedly reported as being very difficult for children with syntactic DLI (Schuele and Nicholls, 2000; Novogrodsky and Friedmann, 2002, 2006; van der Lely and Battell, 2003; Friedmann and Novogrodsky, 2004, 2007, 2008, 2011; Delage et al., 2007; Friedmann et al., 2015). When we only look at percentage correct performance in this task, both groups showed impaired performance. However, the two groups differed substantially in their error types and in the pattern of performance in the different sentence structures. The children with SyDLI showed a clear asymmetry between their production of subject and object relatives, with subject relatives being consistently better produced than object relatives. This was not the case in the ASD group, where only 2 of the 18 children with ASD showed this pattern (the others showed impairment in both subject relatives and object relatives, or even a reversed pattern with normal object relatives and impaired production of subject relatives). The clear difference between the ASD and SyDLI groups was also seen in the errors the two groups produced: the ASD participants, and only them, produced pragmatic errors, which did not occur in the SyDLI group: they failed to respond to the experimenter's question, or provided a response that was unrelated to the picture presented. Similar infelicitous responses were also reported by Modyanova et al. (2017).

Experiment 2, in which we examined reading and paraphrasing of object relatives in comparison to simple sentences, provided the same insight. Indeed, the ASD participants failed on this syntactic task, but their pattern of performance across the sentence types as well as the types of errors indicated that their deficit is very different in nature from the one seen in the participants with SyDLI. The SyDLI group showed a clear difference between the object relatives and the simple sentences in both reading of the homograph and in paraphrasing the sentences. In reading the homographs, the SyDLI participants had very few errors, but when they did, it happened when the homograph was embedded in a relative clause. The ASD group, in contrast, made homograph reading errors on both sentence types. The same pattern was also seen in their paraphrasing of the written sentences: The SyDLI participants found it very difficult to understand and paraphrase the object relatives, but paraphrased the simple sentences very well. The performance of the ASD participants was markedly different: their paraphrasing of both the object relatives and the simple sentences was very poor. Like in Experiment 1, the two groups also differed in their error patterns: whereas the SyDLI participants made thematic role errors in their paraphrases, whereby they confused the agent and the theme of the verbs in the sentence, the ASD participants provided a myriad of unexpected and infelicitous responses that either included a random list of words from the sentence, or a repetition of the sentence with a random change in one of the words, or provided an explanation that had very little to do with the original sentence. These responses were unique to the ASD group and were not seen in either the SyDLI or the TD groups.

In Experiment 3, which examined repetition of various syntactic structures, the performance of the ASD group was poor but, again, very different from that of the SyDLI group. Whereas the SyDLI group struggled with structures with certain types of syntactic movement (Wh movement and verb movement), they showed good performance on all other structures—they repeated well simple sentences, as well as sentences with sentential complements to verbs, and sentences with A-movement in which the object of an unaccusative verb moves to subject position. The ASD group showed a completely different pattern, whereby they failed to repeat all kinds of structures in the test. Like in the previous tasks, here, too, the variability within the ASD group was very large, in line with many studies of ASD (Boucher, 2003; Eigsti and Bennetto, 2009; Kwok et al., 2015; Schaeffer, 2016; Modyanova et al., 2017). In this task, too, the errors the ASD participants produced differed from the errors the SyDLI group produced. The SyDLI group made lexical and structural errors only, but the ASD participants, who produced some structural and lexical errors, also produced many errors we did not see in the other groups, mainly errors of perseveration from a previously repeated sentence. They also produced some responses that indicated they understood the task differently: they often added commentaries to the sentence they repeated, interpreted it, or answered it instead of repeating it.

Thus, our study, in line with recent previous research, raises three main types of reservations against suggesting that the syntactic deficit in ASD is the same one we see in SyDLI. The first relates to the **wide variability** in language skills within the ASD group (Tager-Flusberg, 2006): only some, but definitely not all children with ASD show poor performance in syntactic tasks. This led some researchers who compared ASD to SyDLI to divide their ASD participants into subgroups with and without language impairment (e.g., Roberts et al., 2004; Whitehouse et al., 2008; Modyanova et al., 2017). The two other reservations relate to the nature of the data showing similarities between the two groups: studies arguing for similarities between ASD and SyDLI typically used standardized task scores and did not use error analysis or detailed analysis of the exact types of syntactic structures that are impaired in the two groups. Once the **types of errors** are analyzed, a clear difference emerges between the groups: they commit errors of different kinds, indicating different underlying deficits in the ASD and SyDLI groups. This conclusion regarding the importance of error analysis is in line with studies by Demouy et al. (2011), Riches et al. (2010), Modyanova et al. (2017), and Roberts et al. (2004), who tested syntax in ASD in comparison to DLI using various tasks and reported that even when the ASD and the DLI groups achieved similar scores, they showed different error patterns. The DLI group made mainly syntactic errors, but these errors did not characterize the ASD group, who made many pragmatic errors. Finally, the analysis of **patterns of impairment and sparing**, i.e., the syntactic structures on which the participants fail and those on which they perform normally, yields crucial differences between ASD and SyDLI individuals. Like in our current study, Durrleman et al. (2016), who used a careful design of sentence structures with various syntactic properties concluded that unlike children with SyDLI, children with ASD often show an across-the-board deficit in various sentence structures, including simple sentences. Gavarró and Heshmati (2014) made a similar observation in their study of passive sentence comprehension in ASD: the ASD participants made errors not only on the passive sentences but also on active sentences.

Several conclusions can be drawn from the direct comparison between individuals with SyDLI and individuals with ASD using the same syntactic tasks. First, the fact that a person fails in a syntactic task, as indicated by a low percentage correct in the test, does not mean that this person has a syntactic deficit. Failure in syntactic tasks can arise from failure to understand the task, failure to understand the situation described in sentences, failure to establish a felicitous discourse, among many other reasons. Therefore, a general task score is not enough to establish a syntactic deficit, and an in-depth analysis of response types, error types, and a comparison between performance in different structures, e.g., those that involve a certain syntactic complexity and those that do not—is essential.

Secondly, individuals with ASD show great variability that does not allow for a generalization about the syntactic ability of the whole group. Some individuals with ASD also have syntactic difficulties, but some do not. In the current study, only one of the ASD participants showed a syntactic performance that resembled that of children with SyDLI, and seven ASD participants showed intact comprehension, production, and repetition of sentences derived by syntactic movement, once their pragmatic deviant responses were removed. Thus, we can conclude that poor performance in syntactic tasks still does not indicate a syntactic impairment, and that ASD is not DLI.

## Author contributions

NS and NF created together the research questions and the method of exploring them. NF developed the syntactic tests. NS tested all the ASD children. NF together with Rama Novogrodsky and Iris Fattal who are cited in the paper, tested the SyDLI children. NS and NF together analyzed the data, interpreted the data and wrote the paper.

### Conflict of interest statement

The authors declare that the research was conducted in the absence of any commercial or financial relationships that could be construed as a potential conflict of interest. The handling Editor declared a past co-authorship with NF.

## Footnotes

^1^Notes on the choice of the term SyDLI: **(1) Why we prefer DLI over SLI:** We choose to use the term DLI, developmental language impairment, instead of SLI, specific language impairment, following the need to change expressed in Bishop et al. (2017) and Norbury and Sonuga-Barke (2017), for two reasons: the word “specific”, which contributes the S to SLI was meant to exclude individuals with language deficits who also have other types of impairment, such as ASD. Thus, by definition, children with ASD may never have SLI. The other reason is that the term *DLI* adds the important information that the language deficit is developmental. **(2) Why we prefer SyDLI over DLD:** DLI has subtypes, referring to the language domain that is impaired. There are syntactic, phonological, lexical, and possibly also semantic and pragmatic types of DLI. We believe that this heterogeneity should be reflected in the nomenclature of the DLI group tested. In the current study we focus on syntactic impairment, which we term below “SyDLI”, syntactic DLI. We preferred not to use the term developmental syntactic disorder, DSD, which would be too confusing with ASD; We preferred SyDLI over SyDLD because SyDLI can be pronounced as a single word, unlike SyDLD (see also Bishop et al., 2017, who suggested the term DLD, for arguments for and against preferring disorder over impairment).

^2^A different type of movement of the phrase, termed *A-movement*, involves the movement of the object to subject position. This more local movement is tested in Experiment 3 and discussed in section Experiment 3: Sentence Repetition Task.

^3^In Israel, placement in ASD-specific classes is highly regulated, requiring a diagnosis from a child psychiatrist or clinical psychologist, as well as the agreement of a seven-member multi-disciplinary panel including a pediatrician, an educational psychologist, a social worker who specializes in children with special needs, the chief inspector of special education in the ministry of education, an inspector of regular education, and a representative of the municipal education committee.

^4^In a way, the participants were also IQ-matched, because all the ASD participants had normal IQ—for 13 of the participants, the ASD diagnosis included normal IQ, and for the other 5, for whom we had no report of pre-tested IQ, we had the performance of the picture association task, which was 92–100.

^5^The structure of our argument was the following: the total performance of the ASD group in the syntactic tasks is not different from that of the SyDLI group, but they do differ in error types and in the structures in which they perform poorly. Furthermore, we show that they do not show any differences between syntactic structures, unlike the SyDLI group. Therefore, our main argument is based on lack of difference (ASD vs. DLI; different syntactic structures within the same task), so in order to work against our claim, we did not use correction for multiple comparisons (which would render significant differences not different).

^6^The SyDLI participants made very few reading errors in words other than the homographs in the relative clauses, in a rate that was not different from TD controls, see Friedmann and Novogrodsky, 2007.

^7^Two of the participants we considered to have normal syntax made word order reversal errors in the repetition task. We nevertheless concluded they had intact syntax because they produced many correct relative clauses in all of their paraphrases, even in paraphrases of simple sentences. (One of them even spontaneously produced verb movement to second position in his paraphrases). We suspect that their failure to repeat the sentences accurately may have resulted from the way they understood the task and from their pragmatic deficit rather than from a syntactic difficulty.

## References

[B1] Adams C. (1990). Syntactic comprehension in children with expressive language impairment. Int. J. Lang. Commun. Disord. 25, 149–171. 10.3109/13682829009011971 2206964

[B2] Albert M. L. Sandson J. (1986). Perseveration in aphasia. Cortex 22, 103–115. 10.1016/S0010-9452(86)80035-1 2423294

[B3] American Psychiatric Association (2000). Diagnostic and Statistical Manual of Mental Disorders, 4th Edn Washington, DC: American Psychiatric Association.

[B4] Armon-Lotem S. Avram I. (2005). The autonomous contribution of syntax and pragmatics to the acquisition of the Hebrew definite article, in UG and External Systems: Language, Brain, and Computation (Amsterdam: John Benjamin Publishing Company), 171–183.

[B5] Baddeley A. D. (1997). Human Memory: Theory and Practice. East Sussex, UK: Psychology Press.

[B6] Balaban N. Friedmann N. Ariel M. (2016). The effect of theory of mind impairment on language: referring after right hemisphere damage. Aphasiology 30, 1424–1460. 10.1080/02687038.2015.1137274

[B7] Berman R. A. (1997). Early acquisition of syntax and discourse in Hebrew, in Psycholinguistic Studies in Israel: Language Acquisition, Reading, and Writing, ed Shimron Y. (Jerusalem: Magnes Press), 57–100.

[B8] Biran M. Friedmann N. (2004). SHEMESH: Naming A Hundred Objects. Tel Aviv: Tel Aviv University.

[B9] Biran M. Friedmann N. (2005). From phonological paraphasias to the structure of the phonological output lexicon. Lang. Cogn. Process. 20, 589–616. 10.1080/01690960400005813

[B10] Biran M. Friedmann N. (2007). MA KASHUR: Picture Associations Test. Tel Aviv: Tel Aviv University.

[B11] Biran M. Friedmann N. (2012). The representation of lexical-syntactic information: evidence from syntactic and lexical retrieval impairments in aphasia. Cortex 48, 1103–1127. 10.1016/j.cortex.2011.05.024 21798529

[B12] Bishop D. V. (2001). Genetic and environmental risks for specific language impairment in children. Philos. Trans. R. Soc. Lond. B Biol. Sci. 356, 369–380. 10.1098/rstb.2000.0770 11316485 PMC1088433

[B13] Bishop D. V. (2006). What causes specific language impairment in children? Curr. Dir. Psychol. Sci. 15, 217–221. 10.1111/j.1467-8721.2006.00439.x 19009045 PMC2582396

[B14] Bishop D. V. M. (2003). Autism and specific language impairment: categorical distinction or continuum? in Autism: Neural Basis and Treatment Possibilities (Chichester: Wiley), 213–234. 14521195

[B15] Bishop D. V. M. Snowling M. J. Thompson P. A. Greenhalgh T. the CATALISE-2 consortium (2017). Phase 2 of CATALISE: a multinational and multidisciplinary Delphi consensus study of problems with language development: terminology. J. Child Psychol. Psychiatry 58, 1068–1080. 10.1111/jcpp.12721 28369935 PMC5638113

[B16] Bishop D. (1989). Test for the Reception of Grammar:*(TROG)* Manchester, UK: Medical Research Council.

[B17] Botting N. Conti-Ramsden G. (2003). Autism, primary pragmatic difficulties, and specific language impairment: can we distinguish them using psycholinguistic markers? Dev. Med. Child Neurol. 45, 515–524. 10.1111/j.1469-8749.2003.tb00951.x 12882530

[B18] Boucher J. (2003). Language development in autism. Int. Congress Series 1254, 247–253. 10.1016/S0531-5131(03)00976-2

[B19] Brock J. Sukenik N. Friedmann N. (2017). Individual differences in autistic children's homograph reading: evidence from Hebrew. Autism Dev. Lang. Impair. 2, 1–10. 10.1177/2396941517714945

[B20] Cohen L. Dehaene S. (1998). Competition between past and present: assessment and interpretation of verbal perseverations. Brain 121, 1641–1659. 10.1093/brain/121.9.1641 9762954

[B21] Conti-Ramsden G. Botting N. (1999). Classification of children with specific language impairment: longitudinal considerations. J. Speech Lang. Hear. Res. 42, 1195–1204. 10.1044/jslhr.4205.1195 10515515

[B22] Conti-Ramsden G. Botting N. Faragher B. (2001). Psycholinguistic markers for specific language impairment (SLI). J. Child Psychol. Psychiatry 42, 741–748. 10.1111/1469-7610.00770 11583246

[B23] Conti-Ramsden G. Crutchley A. Botting N. (1997). The extent to which psychometric tests differentiate subgroups of children with SLI. J. Speech Lang. Hear. Res. 40, 765–777. 10.1044/jslhr.4004.765 9263942

[B24] Conti-Ramsden G. Simkin Z. Botting N. (2006). The prevalence of autistic spectrum disorders in adolescents with a history of specific language impairment (SLI). J. Child Psychol. Psychiatry 47, 621–628. 10.1111/j.1469-7610.2005.01584.x 16712639

[B25] Costa J. Friedmann N. (2012). Children acquire unaccusatives and A-movement very early, in The Theta System: Argument Structure at the Interface, eds Everaert M. Marelj M. Siloni T. (Oxford: Oxford University Press), 354–378.

[B26] Crawford J. R. Howell D. C. (1998). Comparing an individual's test score against norms derived from small samples. Clin. Neuropsychol. 12, 482–486. 10.1076/clin.12.4.482.7241

[B27] Creemers A. Schaeffer J. C. (2016). Specific language impairment and high functioning autism: evidence for distinct etiologies and for modularity of grammar and pragmatics, in Proceedings of the 6th Conference on Generative Approaches to Language Acquisition North America (GALANA 2015), eds Perkins L. et al. (Somerville, MA: Cascadilla Proceedings Project), 1–12.

[B28] Delage H. Monjauze C. Hamann C. Tuller L. (2007). Relative clauses in atypical acquisition of French, in Language Acquisition and Development: Proceedings of GALA (Newcastle, UK), 166–176.

[B29] Demouy J. Plaza M. Xavier J. Ringeval F. Chetouani M. Perisse D. (2011). Differential language markers of pathology in autism, pervasive developmental disorder not otherwise specified and specific language impairment. Res. Autism Spect. Disord. 5, 1402–1412. 10.1016/j.rasd.2011.01.026

[B30] Durrleman S. Delage H. (2016). Autism spectrum disorder and specific language impairment: overlaps in syntactic profiles. Lang. Acquist. 23, 361–386. 10.1080/10489223.2016.1179741

[B31] Durrleman S. Delage H. Prévost P. Tuller L. (2017). The comprehension of passives in autism spectrum disorder. Glossa 2, 1–30. 10.5334/gjgl.205

[B32] Durrleman S. Marinis T. Franck J. (2016). Syntactic complexity in the comprehension of wh-questions and relative clauses in typical language development and autism. Appl. Psycholingust. 37, 1501–1527. 10.1017/S0142716416000059

[B33] Eigsti I. M. Bennetto L. (2009). Grammaticality judgments in autism: deviance or delay. J. Child Lang. 36, 999–1021. 10.1017/S0305000909009362 19224652

[B34] Fattal I. Friedmann N. Fattal-Valevski A. (2011). The crucial role of thiamine in the development of syntax and lexical retrieval: a study of infantile thiamine deficiency. Brain 134, 1720–1739. 10.1093/brain/awr068 21558277

[B35] Fattal I. Friedmann N. Fattal-Valevski A. (2013). Language abilities of 8-9 year old children who suffered thiamine-deficiency in infancy, in Presented in the 49th Annual Conference of the Israeli Speech Hearing and Language Association, Tel Aviv.

[B36] Friedmann N. (1998). BAFLA: Friedmann's battery for agrammatism. Tel Aviv: Tel Aviv University.

[B37] Friedmann N. (2000). PETEL: A Sentence Repetition Test. Tel Aviv: Tel Aviv University.

[B38] Friedmann N. (2001). Agrammatism and the psychological reality of the syntactic tree. J. Psycholinguist. Res. 30, 71–90. 10.1023/A:1005256224207 11291184

[B39] Friedmann N. (2007). Young children and A-chains: the acquisition of hebrew unaccusatives. Lang. Acquis. 14, 377–422. 10.1080/10489220701600523

[B40] Friedmann N. Costa J. (2010). The child heard a coordinated sentence and wondered: on children's difficulty in understanding coordination and relative clauses with crossing dependencies. Lingua 120, 1502–1515. 10.1016/j.lingua.2009.10.006

[B41] Friedmann N. Costa J. (2011). Acquisition of SV and VS order in hebrew, European Portuguese, palestinian Arabic, and Spanish. Lang. Acquis. 18, 1–38. 10.1080/10489223.2011.530507

[B42] Friedmann N. Grodzinsky Y. (1997). Tense and agreement in agrammatic production: pruning the syntactic tree. Brain Lang. 56, 397–425. 10.1006/brln.1997.1795 9070419

[B43] Friedmann N. Gvion A. (2003). Sentence comprehension and working memory limitation in aphasia: a dissociation between semantic-syntactic and phonological reactivation. Brain Lang. 86, 23–39. 10.1016/S0093-934X(02)00530-8 12821413

[B44] Friedmann N. Lavi H. (2006). On the order of acquisition of A-movement, Wh-movement and V-C movement, in Language Acquisition and Development, eds Belletti A. Bennati E. Chesi C. Di Domenico E. Ferrari I. (Newcastle: Cambridge Scholars Press), 211–217.

[B45] Friedmann N. Novogrodsky R. (2004). The acquisition of relative clause comprehension in hebrew: a study of SLI and normal development. J. Child Lang. 31, 661–681. 10.1017/S0305000904006269 15612394

[B46] Friedmann N. Novogrodsky R. (2007). Is the movement deficit in syntactic SLI related to traces or to thematic role transfer? Brain Lang. 101, 50–63. 10.1016/j.bandl.2006.09.006 17084444

[B47] Friedmann N. Novogrodsky R. (2008). Subtypes of SLI: SySLI, PhoSLI, LeSLI, and PraSLI, in Language Acquisition and Development, eds Gavarró A. João Freitas M. (Newcastle: Cambridge Scholars Press), 205–217.

[B48] Friedmann N. Novogrodsky R. (2011). Which questions are most difficult to understand?: the comprehension of Wh questions in three subtypes of SLI. Lingua 121, 367–382. 10.1016/j.lingua.2010.10.004

[B49] Friedmann N. Belletti A. Rizzi L. (2009). Relativized relatives: types of intervention in the acquisition of A-bar dependencies. Lingua 119, 67–88. 10.1016/j.lingua.2008.09.002

[B50] Friedmann N. Fattal I. Fattal-Valevski A. (2010). The effect of thiamine deficiency in infancy on the development of syntactic and lexical abilities. Proc.-Soc. Behav. Sci. 6, 168–169. 10.1016/j.sbspro.2010.08.083

[B51] Friedmann N. Szterman R. (2006). Syntactic movement in orally-trained children with hearing impairment. J. Deaf Stud. Deaf Educ. 11, 56–75. 10.1093/deafed/enj002 16192406

[B52] Friedmann N. Yachini M. Szterman R. (2015). Relatively easy relatives: children with syntactic SLI avoid intervention, in Structures, Strategies and Beyond. Studies in Honour of Adriana Belletti, eds Di Domenico E. Hamann C. Matteini S. (Amsterdam: John Benjamins; Linguistik Aktuell series), 303–320.

[B53] Gavarró A. Heshmati Y. (2014). An investigation on the comprehension of Persian passives in typical development and autism. Catal. J. Linguist. 13, 79–98. 10.5565/rev/catjl.151

[B54] Günzberg-Kerbel N. Shvimer L. Friedmann N. (2008). “Take the hen that the cow kissed the hen”: the acquisition of comprehension and production of various relative clauses in hebrew. Lang. Brain 7, 23–43. 10.1016/j.lingua.2010.02.009

[B55] Håkansson G. Hansson K. (2000). Comprehension and production of relative clauses: a comparison between Swedish impaired and unimpaired children. J. Child Lang. 27, 313–333. 10.1017/S0305000900004128 10967890

[B56] Hamann C. (2004). Comparing the development of the nominal and the verbal functional domain in French language impairment, in Language Acquisition and Language Disorders, eds Prévost P. Paradis J. (Amsterdam: John Benjamin Publishing), 109–146.

[B57] Hamann C. (2005). The production of Wh-questions by French children with SLI-movement is difficult, in Presentation at the 10th International Congress for the Study of Child Language, (Berlin: Freie Universität of Berlin).

[B58] Hamann C. Tuller L. (2015). Intervention effects in the spontaneous production of relative clauses in (a) typical language development of French children and adolescents, in Structures, strategies and beyond. Studies in honour of Adriana Belletti, eds Di Domenico E. Hamann C. Matteini S. (Amsterdam: John Benjamins; Linguistik Aktuell series), 321–342.

[B59] Hamann C. Ohayon S. Dubé S. Frauenfelder U. H. Rizzi L. Starke M. (2003). Aspects of grammatical development in young French children with SLI. Dev. Sci. 6, 151–158. 10.1111/1467-7687.00265

[B60] Jakubowicz C. (2011). Measuring derivational complexity: new evidence from typically developing and SLI learners of L1 French. Lingua 121, 339–351. 10.1016/j.lingua.2010.10.006

[B61] Jakubowicz C. Gutierrez J. (2007). Elicited production and comprehension of root wh questions in French and Basque, in Presentation at the Cost Meeting Cross Linguistically Robust Stage of Children's Linguistic Performance, Berlin.

[B62] Jakubowicz C. Tuller L. (2008). Specific language impairment in French, in Studies in French Applied Linguistics, ed Ayoun D. (Amsterdam: John Benjamins), 97–133.

[B63] Jakubowicz C. Nash L. Rigaut C. Gérard C. L. (1998). Determiners and clitic pronouns in French-speaking children with SLI. Lang. Acquis. 7, 113–160. 10.1207/s15327817la0702-4_3

[B64] Jisa H. Reilly J. Verhoeven L. Baruch E. Rosado E. (2002). Passive voice constructions in written text: a crosslinguistic developmental study. Writ. Lang. Lit. 5, 163–182. 10.1075/wll.5.2.03jis

[B65] Kjelgaard M. M. Tager-Flusberg H. (2001). An investigation of language impairment in autism: implications for genetic subgroups. Lang. Cogn. Process. 16, 287–308. 10.1080/01690960042000058 16703115 PMC1460015

[B66] Korkman M. Hakkinen-Rihu P. (1994). A new classification of deamong clinic-referred children. J. Abnorm. Child Psychol. 18, 29–45.

[B67] Kuder G. F. Richardson M. W. (1937). The theory of the estimation of test reliability. Psychometrika 2, 151–160. 10.1007/BF02288391 18145837

[B68] Kwok E. Y. Brown H. M. Smyth R. E. Cardy J. O. (2015). Meta-analysis of receptive and expressive language skills in autism spectrum disorder. Res. Autism Spect. Disord. 9, 202–222. 10.1016/j.rasd.2014.10.008

[B69] Leonard L. B. (2014). Children with Specific Language Impairment. Cambridge, MA: MIT press.

[B70] Leonard L. B. (2017). Reciprocal relations between syntax and tense/agreement morphology in children's interpretation of input: a look at children with specific language impairment. First Lang. 10.1177/0142723717729094

[B71] Linzen T. (2009). Corpus of Blog Postings Collected from the Israblog Website. Tel Aviv: Tel Aviv University.

[B72] Levy H. Friedmann N. (2009). Treatment of syntactic movement in syntactic SLI: a case study. First Lang. 29, 15–49. 10.1177/0142723708097815

[B73] Lloyd H. Paintin K. Botting N. (2006). Performance of children with different types of communication impairment on the clinical evaluation of language fundamentals (CELF). Child Lang. Teach. Ther. 22, 47–67. 10.1191/0265659006ct297oa

[B74] Lombardo M. V. Pierce K. Eyler L. T. Barnes C. C. Ahrens-Barbeau C. Solso S.. (2015). Different functional neural substrates for good and poor language outcome in autism. Neuron 86, 567–577. 10.1016/j.neuron.2015.03.023 25864635 PMC4610713

[B75] Loucas T. Riches N. G. Charman T. Pickles A. Simonoff E. Chandler S.. (2010). Speech perception and phonological short-term memory capacity in language impairment: preliminary evidence from adolescents with specific language impairment (SLI) and autism spectrum disorders (ASD). Int. J. Lang. Commun. Disord. 45, 275–286. 10.3109/13682820902936433 20131963

[B76] Loucas T. Riches N. Baird G. Pickles A. Simonoff E. Chandler S. (2013). Spoken word recognition in adolescents with autism spectrum disorders and specific language impairment. Appl. Psycholinguist. 34, 301–322. 10.1017/S0142716411000701

[B77] Lust B. Flynn S. Foley C. (1996). What children know about what they say: elicited imitation as a research method for assessing children's syntax, in Methods for Assessing Children's Syntax, eds McDaniel D. McKee C. Cairns H. (Cambridge, MA: MIT Press), 55–76.

[B78] Manolitsi M. Botting N. (2011). Language abilities in children with autism and language impairment: using narrative as an additional source of clinical information. Child Lang. Teach. Ther. 27, 39–55. 10.1177/0265659010369991

[B79] McGregor K. K. Berns A. J. Owen A. J. Michels S. A. Duff D. Bahnsen A. J. (2012). Associations between syntax and the lexicon among children with or without ASD and language impairment. J. Autism Dev. Disord. 42, 35–47. 10.1007/s10803-011-1210-4 21365354 PMC3177980

[B80] Modyanova N. Perovic A. Wexler K. (2017). Grammar is differentially impaired in subgroups of autism spectrum disorders: evidence from an investigation of tense marking and morphosyntax. Front. Psychol. 8:320. 10.3389/fpsyg.2017.00320 28400738 PMC5368187

[B81] Norbury C. F. Sonuga-Barke E. (2017). Editorial: new frontiers in the scientific study of developmental language disorders. J. Child Psychol. Psychiatry 58, 1065–1067. 10.1111/jcpp.12821 28921545

[B82] Novogrodsky R. Friedmann N. (2002). Relative clause comprehension in Hebrew-speaking school-age children with G-SLI, in Presented at the Euresco conference The Syntax of Normal and Impaired Language (Corinth).

[B83] Novogrodsky R. Friedmann N. (2006). The production of relative clauses in syntactic SLI: a window to the nature of the impairment. Adv. Speech Lang. Pathol. 8, 364–375. 10.1080/14417040600919496

[B84] Paradis J. Crago M. Genesee F. Beachley B. Brown A. Conlin F. (2003). Object clitics as a clinical marker of SLI in French: evidence from French-English bilingual children, in Proceedings of the 27th Annual Boston University Conference on Language Development, Vol. 2, (Boston, MA), 638–649.

[B85] Parisse C. Maillart C. (2004). Le développement morphosyntaxique des enfants présentant des troubles de développement du langage: Données francophones. Enfance 56, 20–35. 10.3917/enf.561.0020

[B86] Pickles A. Simonoff E. Conti-Ramsden G. Falcaro M. Simkin Z. Charman T.. (2009). Loss of language in early development of autism and specific language impairment. J. Child Psychol. Psychiatry 50, 843–852. 10.1111/j.1469-7610.2008.02032.x 19527315

[B87] Reznick J. Friedmann N. (2017). The order of acquisition of various movement types in hebrew. Language and Brain 12, 109–168.

[B88] Riches N. G. Loucas T. Baird G. Charman T. Simonoff E. (2010). Sentence repetition in adolescents with specific language impairments and autism: an investigation of complex syntax. Int. J. Lang. Commun. Disord. 45, 47–60. 10.3109/13682820802647676 19343567

[B89] Riches N. G. Loucas T. Baird G. Charman T. Simonoff E. (2011). Non-word repetition in adolescents with specific language impairment and autism plus language impairments: a qualitative analysis. J. Commun. Disord. 44, 23–36. 10.1016/j.jcomdis.2010.06.003 20673911

[B90] Roberts J. A. Rice M. L. Tager-Flusberg H. (2004). Tense marking in children with autism. Appl. Psycholinguist. 25, 429–448. 10.1017/S0142716404001201

[B91] Rothweiler M. Chilla S. Clahsen H. (2012). Subject–verb agreement in specific language impairment: a study of monolingual and bilingual German-speaking children. Biling. Lang. Cogn. 15, 39–57. 10.1017/S136672891100037X

[B92] Schaeffer J. (2016). Linguistic and cognitive abilities in children with specific language impairment as compared to children with high-functioning autism. Lang. Acquis. 25, 5–23. 10.1080/10489223.2016.1188928

[B93] Schuele C. M. Nicholls L. M. (2000). Relative clauses: Evidence of continued linguistic vulnerability in children with specific language impairment. Clin. Linguist. Phonet. 14, 563–585. 10.1080/026992000750048116

[B94] Schuele C. M. Tolbert L. (2001). Omissions of obligatory relative markers in children with specific language impairment. Clin. Linguist. Phonet. 15, 257–274. 10.1080/02699200010017805

[B95] Stavrakaki S. (2001). Comprehension of reversible relative clauses in specifically language impaired and normally developing Greek children. Brain Lang. 77, 419–431. 10.1006/brln.2000.2412 11386707

[B96] Stavrakaki S. Chrysomallis M. A. Petraki E. (2011). Subject-verb agreement, object clitics and wh-questions in bilingual French–Greek SLI: the case study of a French-Greek-speaking child with SLI. Clin. Linguist. Phonet. 25, 339–367. 10.3109/02699206.2010.538954 21469971

[B97] Sukenik N. Friedmann N. (2010). Gamad-Gamal: A Word-Picture Matching Test with Phonological and Semantic Distracters. Tel Aviv: Tel Aviv University.

[B98] Szterman R. Friedmann N. (2014). Relative clause reading in hearing impairment: different profiles of syntactic impairment. Front. Psychol. Lang. Sci. 5, 1–16. 10.3389/fpsyg.2014.01229 25426086 PMC4224075

[B99] Szterman R. Friedmann N. (2015). Insights into the syntactic deficit of children with hearing impairment from a sentence repetition task, in Language Acquisition and Development: Generative Approaches to Language Acquisition 2013C, eds Hamann Ruigendijk E. (Newcastle: Cambridge Scholars Publishing), 492–505.

[B100] Tager-Flusberg H. (2006). Defining language phenotypes in autism. Clin. Neurosci. Res. 6, 219–224. 10.1016/j.cnr.2006.06.007

[B101] Terzi A. Marinis T. Kotsopoulou A. Francis K. (2014). Grammatical abilities of Greek-speaking children with autism. Lang. Acquis. 21, 4–44. 10.1080/10489223.2013.855216

[B102] Tomblin B. (2011). Co-morbidity of autism and SLI: kinds, kin and complexity. Int. J. Lang. Commun. Disord. 46, 127–137. 10.1111/j.1460-6984.2011.00017.x 21401812

[B103] Tuller L. Ferré S. Prévost P. Barthez M. A. Malvy J. Bonnet-Brilhault F. (2017). The effect of computational complexity on the acquisition of French by children with ASD, in Innovative Investigations of Language in Autism Spectrum Disorder, ed L. Naigles (Washington, DC: Walter de Gruyter), p. 115–140.

[B104] Tuller L. Prévost P. Morin E. Zebib R. (2011). Formal language impairment in French-speaking children with ASD: a comparative ASD/SLI study, in Advances in Language Acquisition, eds Stavrakaki S. Lalioti M. Konstantinopoulou P. (Newcastle: Cambridge Scholars Publishing), 472–484.

[B105] van Daal J. Verhoeven L. van Balkom H. (2004). Subtypes of severe speech and language impairments: psychometric evidence from 4-year-old children in the Netherlands. J. Speech Lang. Hear. Res. 47, 1411–1423. 1411–1423. 10.1044/1092-4388(2004/105) 15842019

[B106] van der Lely H. K. J. (1996). Specifically language impaired and normally developing children: verbal passive vs. adjectival passive sentence interpretation. Lingua 98, 243–272. 10.1016/0024-3841(95)00044-5

[B107] van der Lely H. K. Battell J. (2003). Wh-movement in children with grammatical SLI: a test of the RDDR hypothesis. Language 79, 153–181. 10.1353/lan.2003.0089

[B108] van der Lely H. K. Harris M. (1990). Comprehension of reversible sentences in specifically language-impaired children. J. Speech Hear. Disord. 55, 101–117. 10.1044/jshd.5501.101 2299827

[B109] Varlokosta S. Armon-Lotem S. (1998). Resumptives and wh-movement in the acquisition of relative clauses in modern Greek and Hebrew, in Proceedings of the 22nd annual Boston University Conference on Language Development, Vol. 73746 Boston, MA.

[B110] Wexler K. (2011). Grammatical computation in the optional infinitive stage, in Handbook of Generative Approaches to Language Acquisition: Studies in Theoretical Psycholinguistics, eds de Villiers J. Roeper T. (The Netherlands: Springer Dordrecht), 53–118.

[B111] Whitehouse A. J. Barry J. G. Bishop D. V. (2008). Further defining the language impairment of autism: is there a specific language impairment subtype? J. Commun. Disord. 41, 319–336. 10.1016/j.jcomdis.2008.01.002 18295779

[B112] Williams D. Payne H. Marshall C. (2013). Non-word repetition impairment in autism and specific language impairment: evidence for distinct underlying cognitive causes. J. Autism Dev. Disord. 43, 404–417. 10.1007/s10803-012-1579-8 22733298

