# Supplementary material for: ASD Is Not DLI: Individuals With Autism and Individuals With Syntactic DLI Show Similar Performance Level in Syntactic Tasks, but Different Error Patterns
Source: Front Psychol. 2018 Apr 4;9:279. doi: 10.3389/fpsyg.2018.00279 (PMC5894483; doi:10.3389/fpsyg.2018.00279)
Supplement: Supplementary file 1 [file DataSheet1.docx]

**Appendix A. Background information about the participants with ASD**

| Participant | Age (year; month) | Gender | Clinical diagnosis | TILTAN nonword reading (%) | SHEMESH picture naming (%) | GAMAD  Word-picture matching (%) | Picture association (%) |
| --- | --- | --- | --- | --- | --- | --- | --- |
| OM | 9;0 | M | ASD | 100 | 73 | 94 | 92 |
| RV | 9;7 | M | HFA | 53 | 92 | 100 | 95 |
| TI | 9;7 | M | HFA | 90 | 81 | 97 | 100 |
| MR | 10;1 | M | ASD | 80 | 88 | 97 | 95 |
| TR | 10;10 | F | HFA | 57 | 85 | 100 | 100 |
| LR | 11;2 | M | HFA | 63 | 89 | 100 | 97 |
| LN | 11;3 | M | ASD | 100 | 96 | 100 | 95 |
| RN | 11;6 | F | HFA | 73 | 89 | 97 | 100 |
| TO | 11;8 | M | HFA | 100 | 86 | 94 | 89 |
| RA | 15;5 | M | ASD | 70 | 94 | 100 | 100 |
| TM | 15;7 | M | HFA | 93 | 95 | 100 | 100 |
| MT | 15;8 | M | ASD | 57 | 97 | 100 | 100 |
| LM | 15;10 | M | HFA | 93 | 100 | 100 | 97 |
| YN | 15;3 | M | HFA | 97 | 95 | 100 | 100 |
| MR | 16;0 | M | HFA | 100 | 92 | 100 | 100 |
| NF | 16;5 | M | HFA | 100 | 94 | 100 | 100 |
| NT | 17;9 | M | HFA | 100 | 94 | 100 | 100 |
| RG | 18;0 | M | HFA | 100 | 96 | 100 | 100 |

Shaded cells indicate performance that is not significantly different from TD controls

**Lexical, reading, and nonverbal assessment measures**

The participants were tested, as part of a larger study, on a battery of language and reading tests, as well as nonverbal conceptual tests.

Clinical Diagnosis: all the participants with ASD in this study were formally diagnosed by a certified practitioner as described in Section 2.1.1. These diagnostic labels were the ones that were reported to us by the schools in which the children with ASD were studying and were all done under the guidelines of the DSM-IV. As per the ethics committee permission we had, we had access only to the final diagnosis but not to the scores of the IQ testing. So for the participants who were diagnosed with high-functioning autism, we only received the information that their IQ was within the normal range. For the five individuals who were diagnosed as ASD (or PDD NOS) there was no IQ information available. We, therefore, tested all of them using a non-verbal conceptual picture association task, as a proxy for non-verbal IQ.

*Non-verbal conceptual picture association test:* The participants' non-verbal conceptual abilities were assessed using the MA KASHUR picture association test (Biran & Friedmann, 2007). This task was aimed at testing the conceptual system, the way the participants understood relations between objects, and their world knowledge. The test includes 35 triads of pictures - a target picture in the top half of a display (e.g., a picture of a bowl of soup), with two additional pictures presented underneath that are conceptually related to each other (e.g., pictures of a spoon and a fork). The participants were requested to decide which of the two pictures was more closely related to the target picture. (Additional examples include: a target picture of long hair -- with a hairbrush and a toothbrush; a target picture of wool with a lion and a sheep. For details see Biran & Friedmann, 2012).

*We also used two lexical tests – one tested lexical input, the other lexical output*

*Word-picture association* (Gamad-Gamal test, Sukenik & Friedmann, 2010) was used to assess the participants' comprehension of written words, when no verbal response was required. The participants were shown a written word along with three pictures, and were asked to read the word aloud and then point to the corresponding picture. The three pictures were the target picture, a semantic distracter, and a phonological distracter. The task included 40 items.

*Picture naming:* Lexical retrieval was assessed using the SHEMESH picture naming test (Biran & Friedmann, 2005). Participants were asked to name 100 colour pictures of objects of various semantic categories (animals, tools, fruit, vegetables, vehicles, musical instruments, utensils, appliances, apparel, jewellery, body parts) and were scored on the proportion of immediate correct responses. The target words were 1-4 syllable nouns. Mean frequency of these nouns is 81 occurrences in a million (SD = 251; range 1 - 2006) based on a corpus of 165 million words from the Israblog website (Linzen, 2009). Reliability is 0.836 based on a sample of 335 participants with and without naming difficulties. Performance of typically-developing children is close to ceiling (mean = 94.1%; SD = 2.3% for 35 12- to 14- year olds; Biran & Friedmann, 2004, 2005).

Finally, we used a nonword reading task to examine the participants' decoding skills and their phonological output abilities.

*Nonword reading:* the non-word reading test is part of the TILTAN reading screening test (Friedmann & Gvion, 2003). The TILTAN has a reliability coefficient of 0.968 (Kuder & Richardson, 1937) based on a sample of 1022 children with and without reading difficulties. It was developed to detect all known types of dyslexia and decoding difficulties, by using items that are sensitive to the various types of errors. Participants read aloud 30 single nonwords (4–6 letters long).
